# Quantitative Synthesis of Personalized Trials Studies: Meta-Analysis of Aggregated Data Versus Individual Patient Data

**DOI:** 10.1162/99608f92.3574f1dc

**Published:** 2022-09-08

**Authors:** Mariola Moeyaert, Joelle Fingerhut

**Affiliations:** 1 University at Albany-SUNY, Department of Educational and Counseling Psychology – 1400 Washington Ave, Albany, NY 12222; 2 Rutgers University, Graduate School of Applied and Professional Psychology, – 152 Frelinghuysen Rd, Piscataway, NJ 08854

**Keywords:** personalized trials, effect size, aggregated data meta-analysis, individual patient data meta-analysis, multilevel modeling, evidence-based decision and inferences

## Abstract

We have entered an era in which scientific knowledge and evidence increasingly inform research practice and policy. As there is an exponential increase in the use of personalized trials, there is a remarkable growing interest in the quantitative synthesis of personalized trials. One technique that is developed and can be applied for this purpose is meta-analysis. Meta-analysis involves the quantitative integration of effect sizes from several personalized trials. In this study, aggregated data (AD) and individual patient data (IPD) methods for meta-analysis of personalized trials are discussed, together with an empirical demonstration using a subset of a real meta-analytic data set. For the empirical demonstration, 26 personalized trials received usual care and yoga intervention in a randomized sequence. Results show a general consensus between the AD and IPD approach in terms of conclusions—that both usual care and the yoga intervention are effective in reducing pain. However, the IPD approach provides more information about the intervention effectiveness and intervention heterogeneity. IPD is a more flexible modeling approach, allowing for a variety of modeling options.

## 1. Quantitative Synthesis of Personalized Trials: Meta-Analysis of Aggregated Data Versus Individual Patient Data Introduction

Personalized trials, also known as single-case experiments, is a research design that measures the dependent variable within one case (i.e., trial) and at different moments in time during baseline (i.e., control) conditions and experimental conditions (n.d.-a). Within the past few decades, a multitude of different statistics have been developed for use with personalized trials. (Manolov & Moeyaert, 2017) provide an overview of statistics that can be used for the quantitative summary of the effectiveness of an intervention using personalized trials. Guidance and recommendations are provided to select the best appropriate statistic given the data characteristics and the research question(s) of interest. Fingerhut et al. (2020) build further upon this framework by introducing and empirically validating a user-friendly point-and-click tool that can assist applied researchers in the process of selecting an appropriate statistic (the tool is available through this link: https://osf.io/7usbj/). In order to make generalizations (i.e., inferences) about intervention effectiveness beyond the individual, the personalized trial is traditionally replicated across multiple individuals (Shadish & Rindskopf, 2007)(n.d.-b). If evidence in support of intervention effectiveness is consistent across trials, one can be confident that there is truly an effect, and that the effect is not caused by an outside experimental event that happened at the time of intervention delivery (Moeyaert et al., 2013). In addition, if the start of the intervention is randomized and staggered across trials, the internal validity of the experiment can be further increased (n.d.-c)(n.d.-d). If multiple intervention conditions are included in the design, then a randomized sequence, counterbalanced across the trials, is required to minimize order effects (n.d.-e).

Traditionally, multiple participants are embedded in a personalized trial study (in order to enhance internal and external validity), and results can also be quantitatively synthesized at the study level (Moeyaert, Ferron, et al., 2014). In order to further generalize conclusions about the effectiveness of the intervention, personalized trial studies can be replicated. Alternatively, a systematic literature search can be conducted to identify personalized trial studies investigating the effectiveness of the same intervention for the same population and measuring the same outcome variable(s). Meta-analytic techniques can be used to quantitatively synthesize research evidence across personalized trial studies (Moeyaert, 2019).

Techniques to meta-analyze personalized trial studies are distinct from traditional meta-analytic techniques for group comparison designs and observational studies (Michael Borenstein et al., 2009). First, the appropriate effect size is dependent on the data and design characteristics of the trial. Second, the individual is repeatedly measured over time, and therefore trends and serial dependency (i.e., autocorrelation, Ferron, 2002; (Petit-Bois et al., 2016)) between consecutive data points are plausible. Third, the individual serves as its own control, and therefore the statistic reflects the intervention effect at the individual level (and not at the group level and/or study level). Because of these challenges, there is a need to introduce meta-analytic techniques that can account for these factors. The focus of this study is to demonstrate the usability of meta-analytic techniques suitable to synthesize effect sizes across personalized trials. Meta-analytic techniques have the potential to contribute to evidence-based decision-making about what intervention is working, and under which circumstances (Moeyaert, Manolov, et al., 2020). In addition, this approach has the potential to provide insights into whether there is consistent evidence across trials, or whether there is a significant amount of variability in intervention effectiveness between trials (i.e., intervention heterogeneity). In case a significant amount of variability is identified, moderators can be added to the meta-analytic model in an effort to explain under which conditions an intervention is most effective (Moeyaert & Yang, 2021). Therefore, more informed recommendations can be made to the field, and resources can be allocated appropriately.

In order to run a meta-analysis, data from personalized trials need to be preprocessed as a summary statistic (i.e., effect size) per trial. This is the equivalent of calculating Cohen’s d (or Hedges’ g) for group comparison design studies. The difference is that for personalized trials, the summary statistic is calculated at the individual level and the researcher needs to select an appropriate statistic as there are a variety of different statistics available. The summary statistic for group design studies is calculated at the study level, and the researcher does not need to make a selection because there is a consensus that Cohen’s d and Hedges’ g are the best suitable statistics. In addition to the summary statistic (reflecting intervention effectiveness), a measure of precision is needed (n.d.-f). In contrast, selecting a summary statistic (and precision) for personalized trials is not straightforward as there are a variety of statistics introduced and recommended to reflect the intervention effect (i.e., summary statistic). Some of these statistics have desirable statistical properties, and have a well-established and known sampling distribution, which is needed to calculate the standard error (i.e., precision). An example of this are the regression-based statistics (Swaminathan et al., 2014). Other statistics, such as the majority of nonoverlapping statistics (Parker et al., 2011), were developed without reference to a sampling distribution and as such are less suitable for quantitative synthesis. Nevertheless, these statistics have been used in meta-analyses as well (Jamshidi et al., 2022). In this study, we will discuss both groups of statistics (i.e., regression-based statistics and nonoverlapping statistics). After introducing these two groups of summary statistics, the aggregated data (AD) meta-analysis and the individual patient data (IPD) meta-analysis will be introduced.

### 1.1. Summary Statistics

#### 1.1.1. Regression-Based Statistics

An ordinary least square (OLS) regression can be used to estimate the change in outcome level between pre- and postintervention data. The resulting regression-based statistic has desirable statistical properties, and its standard error can be obtained. The simplest OLS-modeling approach includes two parameters: an intercept and a dummy variable (${Intervention}_{ij}$) reflecting whether observation *i* from trial (i.e., participant) *j* belongs to the preintervention phase (${Intervention}_{ij}$ = 0) or to the intervention/postintervention phases1^1^ (${Intervention}_{ij}$ = 1):

(1) $$Y_{ij}=\beta_{0j}+\beta_{1j}{Intervention}_{ij}\;{+\;e}_{ij}\;\text{with}\;e_{ij}∼N\left(0,\sigma_{e}^{2}\right)$$

Using Equation 1, $\beta_{0j}$ reflects the outcome level preintervention, and $\beta_{1j}$ reflects the change in outcome level between the baseline and the intervention phase. The estimate of $\beta_{1j}$ and its standard error can be calculated for each of the personalized trials, and used as summary statistic for the meta-analysis. Another statistic, known as the standardized mean difference (SMD; (n.d.-g), is closely related to $\beta_{1j}$. The SMD is essentially the difference between the baseline and intervention mean divided by the pooled standard deviation (or the standard deviation of the baseline condition). The SMD is calculated from the raw data, whereas the regression-based statistic is estimated from the raw data, and as such modeling assumptions are needed.

The OLS regression model can be easily extended by including a parameter reflecting the time trend, ${Time}_{ij}$. For instance, a linear time trend during the preintervention phase can be modeled. In addition, a change in linear time trend between the pre- and postintervention phases can be modeled by creating an interaction between the parameters ${Time}_{ij}$ and ${Intervention}_{ij}$. This results in the following four-parameter piecewise regression model:

(2) $$\begin{array}{rrr} & Y_{ij}=\beta_{0j}+\beta_{1j}{Time}_{ij}\;+\;\beta_{2j}{Intervention}_{ij} & \\ & & {+\;\beta_{3j}{{Time'}_{ij}\times Intervention}_{ij}+\;e}_{ij}\;with\;e_{ij}∼N\left(0,\sigma_{e}^{2}\right) \end{array}$$

In order to give a meaningful interpretation to the regression parameters, ${Time}_{ij}$ is coded from 0 to I (with increments of 1); 0 refers to the first measurement, 1 to the second measurement, and so forth. An equal time interval between measurements is assumed. ${Time'}_{ij}$ indicates that the time of the interaction term is centered around the first measurement of the intervention phase (or postintervention phase). Assume $n_{a}$ and $n_{b}$ represent the number of measurements during the baseline and the intervention condition, respectively. Then ${Time'}_{ij}$ is coded from -$n_{a}$ to −1 during the baseline condition and from 0 to $n_{b-1}$ during the intervention condition. Given this parameterization, $\beta_{2j}$ reflects the immediate effect of the intervention on the outcome level, and $\beta_{3j}$ reflects the change in linear time trend between pre- and postintervention. This indicates that there are two summary statistics suitable to be meta-analyzed depending on the meta-analytic research question. Equation 2 can be extended to account for other functional forms such as quadratic or nonlinear trends. For more information about parameterization and interpretation of regression-based coefficients, see (Moeyaert, Ugille, et al., 2014a).

The OLS approach, which is used in current study, assumes that the within-participant residuals, ${\;e}_{ij}$’s, are homogeneous, independent and normally distributed. If a researcher is not willing to make these strong data assumptions, a generalized least square (GLS) regression can be run, allowing dependency between residuals (i.e., autocorrelation). It is reasonable to assume that error terms closer in time are more related to one another than residuals terms further away in time. Therefore, a GLS with modeling a lag-1 autocorrelation is suitable (Ferron, 2002). It can also be the case that an outcome is not continuous. For instance, if the measurement scale of the outcome variable is dichotomous or of the outcome variable is a count, a logistic model can be run, as investigated by Declercq et al. (2019). Although the issue of autocorrelation and count outcomes have primarily studied in isolation, they can also co-occur, as further studied by (Swan & Pustejovsky, 2018) and (Swan et al., 2020), but further research is needed. For didactical purposes, continuous outcomes and no autocorrelation are assumed in the current study.

The regression-based approach is flexible as variables can be added to reflect more complex personalized trials that have more than one intervention phase, such as reversal designs. The model can also easily be extended to reflect alternating treatment designs, changing criterion designs, or combined designs (see (Moeyaert, Ugille, et al., 2014a) (Moeyaert, Akhmedjanova, et al., 2020).

#### 1.1.2. Nonoverlapping Statistics

A number of nonoverlapping statistics have been developed for use with personalized trials. Percent of nonoverlapping data (PND) was introduced in 1987 and developed by (Scruggs et al., 1987). PND relies upon the highest data point for the calculation, and so PND is highly influenced by outliers. As a result, the percentage of data points exceeding the median (PEM;(Ma, 2006) was developed, which instead relies on the baseline median for the calculation. PEM has some limitations, including that it has low power and has a severe ceiling effect (Brossart et al., 2014). The percentage of all nonoverlapping data (PAND; (Parker et al., 2007), which considers all data points in the baseline phase, was also developed to address the limitation of PND relying on one data point. (Parker et al., 2011)found that PAND discriminates among the lowest 10% of effects, while (Chen et al., 2016) found that it does not discriminate well against the most successful 20% of interventions.

The improvement rate difference (IRD; Parker et al., 2009) is a nonoverlap statistic that is interpreted as the difference in the proportion of improved scores between the baseline and intervention phase. Although IRD demonstrates greater discriminability compared to other statistical measures (Chen et al., 2016), it has large ceiling effects (Chen et al., 2016) and, like the other aforementioned statistical measures, IRD is insensitive to data trend (Brossart et al., 2014). The nonoverlap of all pairs (NAP; (Parker & Vannest, 2009)) is another measure that was developed for used with personalized trials. NAP is advantageous over previously developed statistics because it is directly calculated from the raw scores without the step of ‘minimum data points removal’ (n.d.-h). It has a known sampling distribution. NAP is insensitive to data trend and is not easily calculated by hand due to the more complex formula (n.d.-i).

Tau-U builds upon the original NAP calculation by removing the amount of overlap from the percentage of nonoverlapping data. There are two versions of Tau-U that integrate trend into the formula, addressing the limitations of previous nonoverlap statistics. These can be denoted as Tau-U _Trend A_ (Parker et al., 2011) and baseline corrected Tau-U (Tarlow, 2017). Each of the three Tau-U variants have strengths and weaknesses and should be used in different circumstances (Fingerhut et al., 2021a, 2021b). For details regarding the specific formulas for calculating each of the aforementioned nonoverlapping statistics, readers are recommended to refer to the original papers of the founders, or to comprehensive calculation tools such as the one developed by Fingerhut et al. (2020; https://osf.io/7usbj/).

### 1.2. Synthesis Across Personalized Trials: Meta-Analysis

Different meta-analytic methods can be applied to synthesize data from personalized trials, such as calculation of a simple average, median, or range of summary statistics. Alternatively, more advanced techniques can be applied, such as aggregate data meta-analysis (AD) and individual participant data (IPD) meta-analysis (Burke et al., 2017). See Cooper and Patall (2009) for an introduction into AD and IPD meta-analysis.

#### 1.2.1. Aggregated Data Meta-Analysis

Aggregated data (AD) meta-analysis is the statistical synthesis of effect sizes or other summary or test statistics calculated for individual trials to provide a conclusion about the overall effect (e.g., an estimate of the average effect size). The simplest AD approach results in the simple average, weighted average, or the range of summary statistic. This approach is traditionally used to obtain the overall effect across trials using nonoverlap statistics as summary statistic (because the majority of these statistics do not have a well-establishing sampling distribution). The nonoverlap statistics are calculated using the raw personalized trial data. Instead of using the raw data as input of the meta-analysis, the summary statistics are used. Alternative AD methods involve vote counting (Michael Borenstein et al., 2009) or combining *p* values (Michael Borenstein et al., 2009)(Onghena & Edgington, 2005). Vote counting is the process of counting the number of statistically significant trials versus the number of not significant trials. This procedure is not recommended to be used alone unless none of the trials contain sufficient information for estimating the effect size (n.d.-j). The *p* values of multiple personalized trials testing the same null hypothesis can be aggregated. Heyvaert et al. (2017) combined *p* values based on randomization tests and found that the combined *p* values approach provides a valid test of overall intervention effect.

#### 1.2.2. Individual Participant Data Meta-Analysis

Another meta-analytic method, especially suited for personalized trials, is individual participant data (IPD) meta-analysis, which involves using the raw data from trials as input of the meta-analysis. Multilevel models proposed by (Van den Noortgate & Onghena, 2008) are especially suitable to synthesize raw data from personalized trials. The repeated raw personalized trial data (level 1) is nested within trials (level 2). An overall estimate of the intervention effects across trials is obtained in addition to variability between trials. Instead of calculating the average of $\beta_{0j},\;\beta_{1j}\;\beta_{2j}$ and $\beta_{3j}$ from Equation 2 across trials, a hierarchical linear model (HLM) can be run instead to provide the overall summary statistic across trials with appropriate standard errors. The HLM approach is a straightforward extension of Equations 1 or 2 (depending on the meta-analytic research question) with the inclusion of a second level allowing the parameters to vary between trials. Allowing the parameters to vary instead of keeping them fixed is less restrictive and does not assume that all participants across all trials have the same baseline parameters and intervention effects. Equation 2 is further extended here:

(3) $$\left\{\begin{array}{l} \beta_{0j}=\theta_{0}+u_{0j}\\ \beta_{1j}=\theta_{1}+u_{1j}\\ \beta_{2j}=\theta_{2}+u_{2j}\\ \beta_{3j}=\theta_{3}+u_{3j} \end{array}\right.\;\text{with}\;\left[\begin{array}{c} \begin{array}{c} u_{0j}\\ u_{1j} \end{array}\\ u_{2j}\\ u_{3j} \end{array}\right]∼N\left(\begin{array}{c} \left[\begin{array}{c} 0\\ 0\\ 0\\ 0 \end{array}\right],\left[\begin{array}{c} \begin{array}{c} \sigma_{u_{0}}^{2}\;\;\;\;\;\;\;\;\;\;\;\;\;\;\;\;\;\;\;\;\;\\ \sigma_{u_{1}u_{0}}\;\;\sigma_{u1\;\;\;\;\;\;\;\;\;\;\;\;\;\;}^{2}\\ \sigma_{u2u0\;}\sigma_{u2u1\;\;}\sigma_{u2}^{2} \end{array}\;\;\;\;\;\;\;\;\;\;\;\;\;\\ \sigma_{u3u0\;}\;\sigma_{u3u1\;\;}\sigma_{u3u2\;}\;\sigma_{u3}^{2}\\ \; \end{array}\right] \end{array}\right)$$

The θ’s indicate the fixed effects and reflect the parameters across trials: $\theta_{0}$ is the outcome level at the start of the baseline phase across all trials, $\theta_{1}$ is the linear time trend during the baseline phase across all trials, $\theta_{2}$ reflects the change in baseline and intervention outcome level at the start of the intervention/postintervention phase, and $\theta_{3}$ is the change in linear trend between the phases. The advantage of this model is that parameters reflecting the variability in intervention effectiveness between trials are included (i.e., between participant/trial-variance) in addition to the residual variance within trials (i.e., within-participant variance). The *u*’s in Equation 3 indicate the deviations from the across-trials parameters. These deviations are assumed to be multivariate normally distributed. The between-trial variance of the intervention effects can be reflected by $\sigma_{u2}^{2}$ and $\sigma_{u3}^{2}$, respectively. The parameters $\sigma_{u2}^{2}$ and $\sigma_{u3}^{2}$ provide helpful information about the homogeneity of intervention effects and can provide an answer to the following question: “Is the intervention (reflected by the change in level $\theta_{2}$ and change in slope $\theta_{3}$) consistently effective for all the participants, or is there a lot of variability in its effectiveness (intervention heterogeneity)?”

This approach can include the trial covariates to check whether they moderate the effect of the intervention (Van den Noortgate & Onghena, 2008). Multilevel models can be adapted easily based on the specific meta-analytic data set and the research interests by including covariates on different levels or by including additional levels. If heterogeneity is identified, variables can be added to the model in an effort to explain variability (Moeyaert & Yang, 2021)(Moeyaert et al., 2022)(n.d.-k). In addition, this approach can use all data within and across different phases from all trials instead of just using the average effect size. HLM can handle a variety of complexities such as accounting for time trends, autocorrelation, or heterogeneity of variances (Hu et al., 2021; (Moeyaert, Ugille, et al., 2014b)(Van den Noortgate & Onghena, 2003b)(Van den Noortgate & Onghena, 2003a)(Van den Noortgate & Onghena, 2008). Note that statistics such as Cochran’s Q statistic (M. Borenstein et al., 2009) or the inconsistency index (Higgins et al., 2003) are not appropriate for detecting heterogeneity when combining raw data using HLM. Instead, HLM provides the between-trial variability in the intercept and the intervention effect, in addition to the within-trial variability (see (Moeyaert et al., 2022), for more detailed discussion regarding heterogeneity).

The statistical properties of summary statistics using the HLM approach has been extensively studied in previous methodological research (See: Ferron et al., 2009; and (Moeyaert, Ugille, et al., 2014a)). The studies indicate that HLM results in unbiased and precise estimates of the intervention effects across trials. Ferron et al. (2014) and (Moeyaert et al., 2022) concluded that HLM has sufficient power to identify intervention effects when combining data from as few as four trials for conditions representative for the field of personalized trials. An additional advantage is that the effects can also be standardized, as this might be needed when trials with different outcome scales are included in the meta-analysis (Van den Noortgate & Onghena, 2008). In this study, all the trials that will be synthesized are measured at the same continuous outcome scale, and therefore standardization is not needed.

## 2. Method and Analysis

### 2.1. Empirical Data Set

The AD and IPD meta-analytic methods will be applied to a subset of a meta-analytic data set (n.d.-l) containing data from 26 personalized trials. The personalized trials consist of a preintervention phase (i.e., baseline phase or A phase), followed by three intervention phases: Usual Care intervention, Yoga intervention and Massage intervention. For demonstration purposes, we only considered two intervention phases: Usual Care intervention and Yoga intervention, and we merged the data across similar conditions. Consequently, conclusions based on our empirical illustrations should only be considered in context of this compromised data set, which is a simplification of the larger meta-analytic data set. The results cannot be used to make recommendations to the field. The sequence of the intervention phases was randomized and counterbalanced across trials (i.e., 13 trials started with the Usual Care intervention whereas the remaining 13 trials started with the Yoga intervention) as can be seen in the visual display in Appendix A. Therefore, the order of intervention administration is not a confounder. The outcome variable is a continuous variable reflecting the pain intensity. The researchers’ interest is whether Yoga practice significantly reduces pain intensity across the trials. In addition, intervention heterogeneity is of interest. The researcher is also interested in whether Yoga practice is significantly more effective in reducing pain relative to Usual Care. In order to investigate this, variables need to be coded accordingly. Two variable identifiers are needed: one reflecting the session (i.e., repeated measures at level one) and one reflecting the trials/participants (level 2). The variable indicating the trials is labeled as ‘Id,’ and the variable indicating the session is labeled as ‘Time.’ Next, two dummy coded variables are needed indicating the phase a session belongs to (i.e., baseline phase, Yoga intervention phase or Usual Care phase). The variable ‘Yoga’ is coded as 1 if a session belongs to the Yoga intervention phase, and the variable ‘CAU’ is coded as 1 if a session belongs to the Usual Care intervention phase. If both variables are coded as 0, then this indicates that the session belongs to the baseline phase. The variable ‘Pain_Intensity_Summary’ is the continuous outcome variable. The raw meta-analytic data set including these variables is available through https://osf.io/ksfe6/. This allows the reader to understand how the data need to be formatted, and to repeat the analyses.

### 2.2. Research Questions and Analytic Approaches

The personalized trial designs included in the meta-analytic dataset are ABC or ACB designs, with A indicating the baseline phase, B indicating the Usual Care phase, and C the Yoga intervention phase. A visual display of the personalized trials included in the meta-analytic data set is displayed in Appendix A.

Using this meta-analytic data set, the following research questions will be investigated:

1. Across all personalized trials, is Yoga practice significantly reducing pain severity?
2. Is there heterogeneity in the effectiveness of Yoga practice between personalized trials?
3. Is Yoga more effective in reducing pain severity symptoms compared to Usual Care?

In order to answer these research questions, the AD and IPD meta-analytic approaches introduced earlier will be applied. The AD is applied to a selection of nonoverlap statistics and the standardized mean difference. Note that a variety of other statistics are available, such as the log response ratio, the regression-based statistic, and the percentage of goal obtained (see Fingerhut et al., 2020). The mean, median, and range of the following summary statistics will be calculated: PND, PEM, IRD, NAP, PAND, Tau-U, and SMD. The R package SingleCaseES v0.4.3 (n.d.-m) is used for the calculations. In addition, the IPD approach, using hierarchical linear modeling of raw personalized data, is applied. Using the IPD approach, data complexities such as linear trends and multiple intervention phases (i.e., Usual Care and Yoga) can be modeled. In addition, estimates for variability in intervention effectiveness between trials can be obtained. The statistical computing environment SAS 9.4 (Copyright © 2015, SAS Institute Inc.) is used for this purpose, and the code can be obtained by contacting the first author of this study.

## 3. Results

### 3.1. Descriptive Statistics

Before discussing the results obtained by the AD and IPD meta-analysis, descriptive statistics are provided in order to have a good understanding of the data at hand. A visual display of the raw data for the 26 personalized trials is provided in Appendix A.

The mean number of total data points for the 26 personalized trials is 60.38 (*Min* = 45, *Max* = 69, *Mdn* = 62, *SD* = 7.18). The mean number of data points during baseline is 13.22 (*Min* = 10, *Max* = 14, *Mdn* = 14, *SD* = 1.21). There are, on average, more data points in the intervention phases than in the baseline phase. The mean number of data points during Usual Care is 24.07 (*Min* = 16, *Max* = 28, *Mdn* = 25, *SD* = 3.47). Similarly, the mean number of data points during Yoga is 24.39 (*Min* = 13, *Max* = 28, *Mdn* = 25, *SD* = 4.15).

The pain intensity score will be the outcome variable of interest in our empirical demonstrations. Therefore, we descriptively describe this outcome variable. The mean pain intensity score during baseline for the 26 personalized trials is 8.38 (*Min* = 3, *Max* = 14, *Mdn* = 9, *SD* = 1.78). The mean pain intensity score during the intervention phases is lower than the mean pain intensity score during baseline phase. The mean pain intensity score during Usual Care is 7.31 (*Min* = 3.00, *Max* = 13, *Mdn* = 7, *SD* = 1.81), while the mean pain intensity score during Yoga is slightly less, at 7.08 (*Min* = 3, *Max* = 12, *Mdn* = 7, *SD* = 1.64).

### 3.2. Aggregated Data Meta-Analysis

Summary Table 1 displays the aggregated summary statistics across the 26 personalized trials. The results are displayed separately for the baseline versus Usual Care comparison, the baseline versus Yoga comparison, and for the Usual Care versus Yoga comparison. Using AD meta-analysis, there is no formal test to investigate whether baseline-Usual Care is significantly smaller compared to the baseline-Yoga intervention comparison. As aggregated summary statistics, the mean, median, and range are reported. Boxplots (Appendix B) are created to visualize the distribution of the summary statistics.

Referring to Table 1, the change between baseline-Yoga is slightly larger than the change between baseline-Usual Care. PND has the lowest mean scores, with 0.28 for baseline-Usual Care, 0.33 for baseline-Yoga, and 0.03 for Usual Care-Yoga (with a positive value indicating that Yoga scores are larger than Usual Care scores). Tau-U has the largest range of scores, ranging from −0.38–1.00 for baseline-Usual Care, −0.17–1.10 for baseline-Yoga, and −0.56–0.71 for Usual Care-Yoga (with a positive value indicating that Yoga scores are larger than Usual Care scores). Referring to Table 1, the SMD score for baseline-Usual Care is 1.42 and 1.45 for baseline–Yoga. Obtaining a score larger than 1.00 is possible as SMD can exceed 1.00, unlike most nonoverlap statistics, which are bound between −1 and 1. An SMD score over 1 can be considered large (Cohen, 1988), and so the SMD scores indicate a large effect for both interventions compared to baseline.

There are currently no firm benchmarks to interpret nonoverlap statistics, as all nonoverlap scores are recommended to be interpreted in context of the data (Vannest et al., 2018). Considering the data for this study, the data appears to be highly variable for many of the trials (see Appendix A). Data variability indicates that the scores of statistics such as nonoverlaps may be highly impacted. This is because by nature, nonoverlap statistics reflect the amount of nonoverlapping data. When there is large data variability, it can be expected that there is a large amount of data overlap. As a result, the nonoverlap scores must be interpreted carefully and within context. The method called Critical Tau can be used (see Fingerhut et al., 2021a) for interpreting the Tau-U results. Critical Tau considers the data characteristics to determine the lowest Tau-U score for which it can be determined there is still evidence of an effect (with a significance level of .05). Using the Critical Tau table located in Appendix A of Fingerhut et al. (2021a), the condition that most closely matches the 26 graphs from Appendix C is as follows: measurement occasions = 40, number of trials = 7, slope = 0, and between-trial variance = 1.00. Thus, the Critical Tau equals 0.536; any Tau-U score above 0.54 likely indicates an effect. Although baseline-Yoga is a little less than 0.54 (as the mean is 0.51), the median is 0.55. This indicates that there may be an effect of Yoga, especially considering that the Critical Tau is a conservative measure. The other nonoverlap statistics (e.g., PEM, PAND, etc.) do not have pre-established benchmarks, making it harder to interpret the baseline-Usual Care and baseline-Yoga scores. The mean and median scores are mostly between 0.40 to 0.70 for both baseline-Usual Care and baseline-Yoga. These scores are above zero; interpreting these scores along with the interpretation of the other scores that show likelihood of an effect (i.e., Critical Tau and SMD), these nonoverlap scores can be interpreted as likely demonstrating that there is at least a small intervention effect (if not medium) for both baseline-Usual Care and baseline-Yoga.

Referring to Table 2, the scores for Yoga are slightly higher than the scores for Usual Care, which may indicate that Yoga is more effective. However, the mean Tau-U score for Usual Care-Yoga is 0.09, which is far below the Critical Tau of 0.54, and so it would be concluded that the Tau-U score indicates there is no change between the two interventions. Similarly, the mean SMD score for this comparison is 0.12, indicating a very small effect (Cohen, 1988). The nonoverlap scores vary around 0.20 to 0.60. This indicates that there may be a positive increase in intervention effectiveness between Usual Care and Yoga.

### 3.3. Individual Participant Data Meta-Analysis

In contrast to the AD meta-analysis, one statistical model can be run to estimate the baseline-Usual Care and baseline-Yoga comparison. In addition, a model can be specified to investigate whether these comparisons are statistically significant. Using this approach, an estimate for the between-trial variability in intervention effectiveness can be obtained. First, an IPD model with only one intervention variable, ‘Yoga,’ will be discussed (Model 1). Next, this model will be extended by including a linear time trend in the baseline, and change in time trends between the baseline and the Yoga intervention phase (Model 2). Lastly a third model will be discussed including the two intervention variables. Model 3a parameterized the model in a way to obtain an estimate of baseline-Usual Care and baseline-Yoga, whereas Model 3b is set up to obtain an estimate of baseline-Usual Care, and Usual Care-Yoga. By using this latter model, it can be investigated whether Yoga is statistically significantly more effective in reducing pain relative to Usual Care. The specific equations are included in Appendix C. The results for the models are displayed in Table 2.

Referring to Model 1, the average baseline level is 8.37, *t*(25.5) = 34.46, *p* < .001. Yoga as an intervention is statistically significantly effective, reducing the pain level from 8.37 to 7.01, $\beta_{1}$ = −1.36, *t*(25.5) = −7.56, *p* < .001. There is a statistically significant amount of between-trial variance for baseline level, $\sigma_{u_{0}}^{2}$= 1.41, *p* <.001. There is also a statistically significant amount of between-trial variance for baseline – Yoga, $\sigma_{u_{1}}^{2}$= 0.64, *p* < .01. The within-trial residual variance is also statistically significant, $\sigma_{e}^{2}$ = 1.65, *p* < .001. Taken together, this between-trial variance indicates that predictors could be added to the second level to explain some of the variability between trials (see (Moeyaert et al., 2022)). However, this is beyond the scope of current study.

The average baseline level for Model 2 is similar to that of Model 1, $\beta_{0}$ = 8.29, *t*(33.3) = 31.35, *p* < .001. There is not a statistically significant time trend in the baseline phase, $\beta_{1}$ = 0.01, *t*(88.8) = 0.92, *p* = .36, indicating that the baseline level remains stable. Yoga as an intervention is statistically significantly effective, reducing the pain level from 8.29 to 7.51, $\beta_{2}$ = −0.78, *t*(38.8) = −3.26, *p* < .01. There is also a statistically significant change in trend between baseline and Yoga, $\beta_{3}$ = −0.08, *t*(88.9) = −3.52, *p* < .001. This indicates that Yoga practice becomes more effective across time. There is a statistically significant amount of between-trial variance for baseline, $\sigma_{u_{0}}^{2}$= 1.45, *p* <.001, and baseline-Yoga (i.e., the immediate effect), $\sigma_{u_{2}}^{2}$= .84, *p* < .01. There is not a statistically significant amount of between-trial variance for time, $\sigma_{u_{1}}^{2}$= 0, *p* = .49, but a statistically significant amount of between-trial variance for change in trend between baseline and Yoga, $\sigma_{u_{3}}^{2}$= 0.004, *p* < .05. There is also a statistically significant amount of within-trial residual variance, $\sigma_{e}^{2}$ =1.40, *p* < .001

The average baseline level for both Model 3a is similar to that of Models 1 and 2, $\beta_{0}$ = 8.38, *t*(26.2) = 34.99, *p* < .001. Usual care is a statistically significantly effective intervention, with participants reporting an average pain level of 7.24 after Usual Care, $\beta_{2}$ = −1.15, *t*(26.6) = −6.45, *p* < .001. Yoga is a statistically significant intervention, with participants reporting an average pain level of 7.02 after Yoga, $\beta_{1}$ = −1.36, *t*(26.3) = −8.16, *p* = .36. There is a statistically significant amount of between-trial variance for baseline average ($\sigma_{u_{0}}^{2}$= 1.36, *p* < .001), baseline-Yoga ($\sigma_{u_{1}}^{2}$= 0.52, *p* < .01), and baseline-Usual Care ($\sigma_{u_{2}}^{2}$= 0.62, *p* < .01), indicating that other predictors can be added to the model to help explain some of the between-trial variability. There is also a statistically significant amount of within-trial residual variance, $\sigma_{e}^{2}$ =1.70, *p* < .001.

The average baseline level for Model 3b is the same as Model 3a, $\beta_{0}$ = 8.38, *t*(25.6) = 33.75, *p* < .001. Same as Model 3a, Usual Care is a statistically significantly effective intervention, with participants reporting an average pain level of 7.24 after Usual Care, $\beta_{1}$ = −1.15, *t*(26.1) = −6.25, *p* < .001. However, the change between Usual Care and Yoga is not statistically significant, $\beta_{2}$ = −0.22, *t*(24.6) = −1.37, *p* = .18, indicating that Yoga is not statistically significantly more effective compared to Usual Care. There is a statistically significant amount of between-trial variance for baseline ($\sigma_{u_{0}}^{2}$= 1.47, *p* <.001), baseline-Usual Care ($\sigma_{u_{1}}^{2}$= 0.68, *p* < .01), and Usual Care-Yoga ($\sigma_{u_{2}}^{2}$= 0.50, *p* < .01). The within-trial residual is also statistically significant, $\sigma_{e}^{2}$ = 1.70, *p* < .001. The statistically significant between-trial variance indicates that there are other predictors that can be added to the model to help explain some of the between-trial variability.

## 4. Discussion

### 4.1. Overall Summary of the Results

The purpose of this demonstration was to show the capabilities of both AD and IPD meta-analytic techniques to synthesize individual trials data. The drawbacks and limitations of AD and IPD approaches are highlighted through application to a data set of 26 personalized trials, where individuals rated pain intensity both before and after receiving Usual Care and a Yoga intervention. Both nonoverlap and standardized mean difference measures were applied to demonstrate the AD approach, while hierarchical linear modeling was used to demonstrate the IPD approach. AD and IPD approaches were used to answer the research questions for this study, which were as follows:

1. Across all personalized trials, is Yoga practice significantly reducing pain severity?
2. Is there heterogeneity in the effectiveness of Yoga practice between personalized trials?
3. Is Yoga more effective in reducing pain severity symptoms compared to Usual Care?

Referring to research question 1, the results of both AD and IPD statistics show that Yoga practice reduces pain severity. While most of the nonoverlap statistics are bound between −1 and 1, they do not have the same benchmarks and the scores need to be interpreted carefully and in context. Although there are no set benchmarks, many of the mean and median nonoverlap scores are between around .50 to .70. Referring to Appendix A, the trial data appears to be highly variable, which may explain why many of the nonoverlap scores were within .50 to .70 and not higher. Thus, this indicates likeliness of an effect, considering the large data variability and assuming that a small reduction in pain intensity indicates the intervention to be practically effective. The conclusion of intervention effectiveness is supported by the other results as well. The Critical Tau-U method indicates there may be an effect. The mean SMD for baseline-Yoga is 1.45. Although the scale might not be applicable to personalized trials, Cohen (1988) recommends for social sciences that SMD = 0.20 indicates a small effect, SMD = 0.50 indicates a median effect, and SMD = 0.80, indicates a large effect. Thus, the SMD score may indicate a very large effect. Referring to the IPD approach, across all three models of hierarchical linear modeling, Yoga was found to be a statistically significantly effective intervention. Hierarchical linear modeling is able to show the average change in level between baseline and intervention. The average change in level parameter is a straightforward outcome and easy to interpret (i.e., Model 1 indicates that Yoga causes an average change in pain intensity of −1.36). This average change in level outcome can be interpreted within context, and the researcher can consider the target population to ultimately determine whether a reduction of 1.36 in pain intensity is practically significant or not. Such ease of interpretation is not provided with the AD method using the mean, median or range of non-overlap statistics.

Answering research question 2 requires the between-trial variability to be estimated so that the homogeneity of the effectiveness can be assessed. The AD statistics, particularly the nonoverlap statistics, are not able to provide an estimate of the heterogeneity between personalized trials. In order to evaluate the heterogeneity while using the AD approach, boxplots can be generated, and the variability can be inspected visually. However, visual inspection can be subjective. The IPD approach using hierarchical linear modeling is able to provide insight into whether heterogeneity is present, and the degree of heterogeneity between personalized trials. The results of this study show that there is significant heterogeneity across personalized trials. This ultimately indicates that other predictors could be added to the models to explain the between-trial differences. Model 2 demonstrates that some of the differences between trials can be contributed to time. When time and change in time between baseline and intervention are added to Model 2, the effect of the Yoga intervention drops from −1.36 in Model 1 to −0.78. This is because some of the effectiveness in Model 1 that was attributed to Yoga is instead attributed to change in trend, as this is a statistically significant predictor in Model 2 ($\beta_{3j}$= .08, *p* < .001). When time is included in the model, it is one moderating factor that may explain some of the differences between personalized trials. The random effects of time and change in time trend are not statistically significant, indicating that the average participant similarly improves across time from receiving the Yoga intervention. Examples of other predictors that could be added to explain the difference between trials include age or gender- perhaps Yoga is more or less effective for different genders or people of different ages. The AD approach is not able to provide objective information regarding heterogeneity; the heterogeneity could be visually inspected in boxplots, and if there is evidence for heterogeneity, different boxplots per level of the moderator could be generated and examined. However, this is time consuming and no actual values are obtained.

Referring to research question 3, it is difficult to determine whether Yoga is more effective than Usual Care using the AD method. Although there is an increase in scores between Usual Care and Yoga across the non-overlap statistics (indicating the possibility that Yoga is more effective than Usual Care), this conclusion is limited by the fact that there are no set benchmarks for interpreting the scores. It is possible that the larger scores for Yoga compared to Usual Care are not practically or statistically significant. The lack of interpretable benchmarks also makes it difficult to interpret the Usual Care-Yoga non-overlap scores with confidence. The Critical Tau indicates there is likely no effect (because the mean Tau-U score of .09 is less than Critical Tau of .054). Similarly, the SMD mean score indicates that there is likely no difference (or a very small difference) between the two interventions (*d* = 0.12; Cohen, 1988). While SMD scores are easier to interpret, it is not possible to test for statistical significance. Using the IPD method of hierarchical linear modeling, the change between Usual Care and Yoga can be formally tested and estimated. This is what is tested in Model 3b. Model 3b shows the average difference in pain intensity between Usual Care and Yoga to be −0.22, but this change is not statistically significant (*p* = .18); there is not a statistically significant difference in effectiveness between Usual Care and Yoga. Overall, considering that HLM and the more easily interpretable AD statistics (Tau-U with the Critical Tau-U and SMD) indicate that there is a very small or no effect, this is likely the accurate conclusion, but more research is needed to determine the true differences in effectiveness between Usual Care and Yoga.

### 4.2. Implications and Recommendations

The current study demonstrates the differences in information provided by the AD and IPD meta-analytic approaches. The IPD approach can be beneficial for learning more in-depth information about intervention effectiveness, and for whom the intervention is effective. The AD method is not able to take the nested structure of data into account and is thus unable to provide an estimate regarding between-trial differences. The estimation of heterogeneity is helpful for determining whether there are differences between groups of people concerning intervention effectiveness. For example, this study shows that change in trend between baseline and intervention is a significant predictor explaining some differences between trials, and that adding other predictors to the models, such as gender or age, may help further identify differences between individuals and intervention effectiveness. Hierarchical linear modeling is able to determine if intervention heterogeneity is present, and this information can help determine whether further moderators should be explored. By using the AD approach, it is not possible to determine whether the interventions are more or less effective for certain groups of people. IPD allows more details to emerge about the interventions and for whom the interventions are effective, which is helpful for applied research and policymaking.

Another advantage of the IPD method is that it allows for time components to be modeled. Adding time components to the model helps to determine if the outcome level changes across time, and if the intervention becomes more or less effective over time. In addition, it can be evaluated whether similar data patterns are obtained in the baseline condition compared to the intervention condition (e.g., the change in linear time trend between baseline and intervention). In the empirical illustration, there is a statistically significant change in trend, telling the researchers that the linear time trend during the intervention phase is statistically significantly different compared to the linear time trend during the baseline. This information cannot be obtained from the AD approach, which does not allow for the modeling of time components. The IPD approach also allows the researcher to determine if the overall effect is statistically significant; statistical significance is widely used to demonstrate intervention effectiveness and is information that can easily be understood and interpreted by researchers across fields. Furthermore, the magnitude of the intervention effect on the original scale is obtained. Thus, the IPD approach reflects the intervention effect on the original scale, which is helpful for evaluating the practical effectiveness.

It is also worth noting that the AD approach can be more time-consuming than the IPD approach, as separate calculations need to be performed for each intervention comparison. In this demonstration, an ABC design was utilized. Using the AD approach, an A-B comparison and then A-C comparison need to be conducted separately. Using the IPD approach, these calculations can be done at the same time and incorporated within the same model (e.g., see Model 3a in Appendix C). Using the IPD method allows for flexibility, customization, and the addition of moderators depending on what the research questions are.

There are several issues with nonoverlap indices and the interpretations of outcomes. Nonoverlap indices do not reflect the size of the effect on an original scale, limiting the interpretation in terms of what can be considered small, medium, or large effects. Although nonoverlap statistic scores are often interpreted using benchmarks (e.g., (Vannest & Ninci, 2015) for NAP; (Scruggs et al., 1987) for PND), it is important that nonoverlap scores are interpreted in context (Vannest et al., 2018), and so in this demonstration we avoid using these stringent benchmarks and instead consider the data characteristics and the dependent variable being measured when interpreting scores. As a result, it is more difficult to reach a firm conclusion when using nonoverlap statistics. Another issue with interpretation is that these nonoverlap indices are believed to be bound within −1 and 1, but this is not accurate. For example, within this study, the Tau-U upper range for Yoga is 1.10, showing how sometimes the scores from this statistic can exceed 1.00. This further highlights the issue with interpreting nonoverlap indices. It can be difficult to truly know the size of the effect and what the nonoverlap score truly represents.

### 4.3. Limitations and Future Directions

This study applied the AD and IPD method to an ABC design to show the benefits and drawbacks of each approach. AD and IPD approaches can be applied to other types of personalized trials designs, such as withdrawal or multiple-baseline design. (Moeyaert et al., 2015) demonstrate how intervention effects can be estimated across different design types. This article focused on an intervention that had a small effect with large variability in many of the subjects (see graphs in Appendix A). It is likely that the nonoverlap results would be more consistent and easier to interpret if the data had a larger effect and less variability (Fingerhut et al., 2021a). However, a real data set was used, showing the ‘messiness’ and careful consideration that occurs when interpreting effects of personalized trials.

This demonstration article only highlighted a few different AD and IPD approaches, and there are others that could be used as well. For example, there are other types of statistics such as the AD approach log-response (Pustejovsky, 2018), or the between-trial standardized mean difference (Pustejovsky et al., 2014), which is an IPD approach. Future research and demonstrations can be done to show the benefits and drawbacks of other types of approaches, for example, comparing certain IPD approaches to each other. It is also important to note that the IPD approach makes some assumptions. For example, it assumes that residuals are multivariate normally distributed, and that the outcome variable is continuous (Moeyaert, Ferron, et al., 2014). If these assumptions are not met, the outcomes may be biased. However, these assumptions can be evaluated prior to analyses with tests such as the Shapiro-Wilk and Kolmogorov-Smirnov. Furthermore, considering several different models with the same conclusions can increase confidence in conclusions made (Moeyaert, Ferron, et al., 2014). Lastly, personalized trials are characterized by a relatively small number of repeated measures and traditionally there are only a limited number of similar trials available for synthesis. Therefore, researchers might consider exploring Bayesian estimation techniques (see (Miočević et al., 2020)and (Moeyaert et al., 2017)).

## Supplementary Material

Appendix B

Appendix A

Appendix C

## Figures and Tables

**Table 1.** Overview of aggregated summary statistics using AD meta-analysis.

	PND	PEM	IRD	NAP	PAND	TauU	SMD
Baseline - Yoga							
Mean	0.33	0.79	0.50	0.75	0.77	0.51	1.45
Median	0.21	0.88	0.47	0.77	0.76	0.55	1.08
Baseline – UC							
Mean	0.28	0.72	0.45	0.71	0.75	0.41	1.42
Median	0.20	0.74	0.36	0.75	0.71	0.48	0.97
UC-Yoga							
Mean	0.03	0.54	0.21	0.55	0.61	0.09	0.12
Median	0.00	0.54	0.17	0.56	0.59	0.12	0.21

*Note.* UC = Usual Care

**Table 2.** Overview of IPD meta-analyses

	Parameter Notation	Parameter Estimate	Standard Error	*p*-value
Model 1				
Fixed Effects				
Pain level baseline	$\beta_{0j}$	8.37*	0.24	<.0001
Baseline-Yoga	$\beta_{1j}$	−1.36*	0.18	<.0001
Random Effects				
Pain level baseline	$\sigma_{u_{0}}^{2}$	1.41*	0.43	.0005
Baseline-Yoga	$\sigma_{u_{1}}^{2}$	0.64*	0.24	.0033
Within-trial residual variance	$\sigma_{e}^{2}$	1.65*	0.08	<.0001
Model 2				
Fixed Effects				
Pain level baseline	$\beta_{0j}$	8.29*	0.26	<.0001
Time baseline	$\beta_{1j}$	0.014	0.016	.3623
Baseline-Yoga (Immediate Effect)	$\beta_{2j}$	−0.78*	0.24	.0023
Baseline-Yoga (Trend effect)	$\beta_{3j}$	−0.075*	0.02	.0007
Random Effects				
Pain level baseline	$\sigma_{u_{0}}^{2}$	1.45*	0.44	.0005
Time baseline	$\sigma_{u_{1}}^{2}$	<0.000023	0.000072	.49
Baseline-Yoga (Immediate Effect)	$\sigma_{u_{2}}^{2}$	0.84*	0.34	.0059
Baseline-Yoga (Trend effect)	$\sigma_{u_{3}}^{2}$	0.0040	0.0018	.0124
Within-trial residual variance	$\sigma_{e}^{2}$	1.40*	0.07	<.0001
Model 3a				
Fixed Effects				
Pain level baseline	$\beta_{0j}$	8.38*	0.24	<.0001
Baseline-Yoga	$\beta_{1j}$	−1.36*	0.17	<.0001
Baseline-Usual Care	$\beta_{2j}$	−1.15*	0.18	<.0001
Random Effects				
Pain level baseline	$\sigma_{u_{0}}^{2}$	1.36*	0.41	.0005
Baseline-Yoga	$\sigma_{u_{1}}^{2}$	0.52*	0.20	.0045
Baseline-Usual Care	$\sigma_{u_{2}}^{2}$	0.62*	0.23	.0029
Within-trial residual variance	$\sigma_{e}^{2}$	1.70*	0.06	<.0001
Model 3b				
Fixed Effects				
Pain level baseline	$\beta_{0j}$	8.38*	0.25	<.0001
Baseline-Usual Care	$\beta_{1j}$	−1.15*	0.18	<.0001
Usual Care-Yoga	$\beta_{2j}$	−0.22	0.16	.1827
Random Effects				
Pain level baseline	$\sigma_{u_{0}}^{2}$	1.47*	0.45	.0005
Baseline-Usual Care	$\sigma_{u_{1}}^{2}$	0.68*	0.24	.0026
Usual Care-Yoga	$\sigma_{u_{2}}^{2}$	0.50*	0.18	.0032
Within-trial residual variance	$\sigma_{e}^{2}$	1.70*	0.06	<.0001

## Footnotes

1 Preintervention is a synonym for baseline phase. The intervention and postintervention reflect the intervention phase. It can be the case that an intervention is delivered, and participants are observed after the intervention delivery. Therefore, these postintervention observations reflect the intervention phase.
