# Supplementary figures and images for: Quantitative Synthesis of Personalized Trials Studies: Meta-Analysis of Aggregated Data Versus Individual Patient Data
Source: Harv Data Sci Rev. Author manuscript; Available in PMC 2023 Nov 24. (PMC10673630; doi:10.1162/99608f92.3574f1dc)

## Appendix B

a.

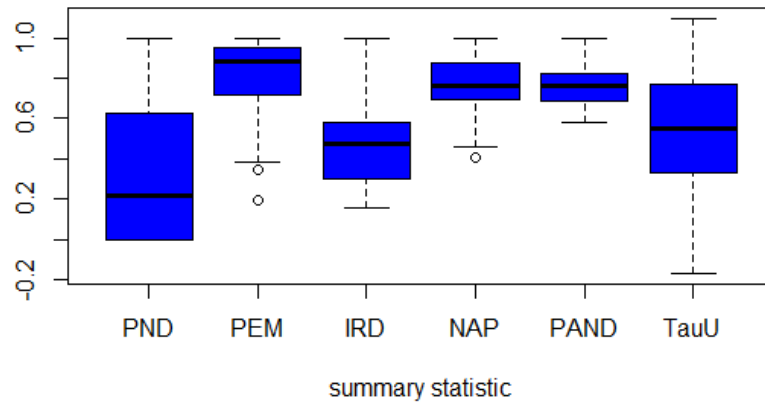

b.

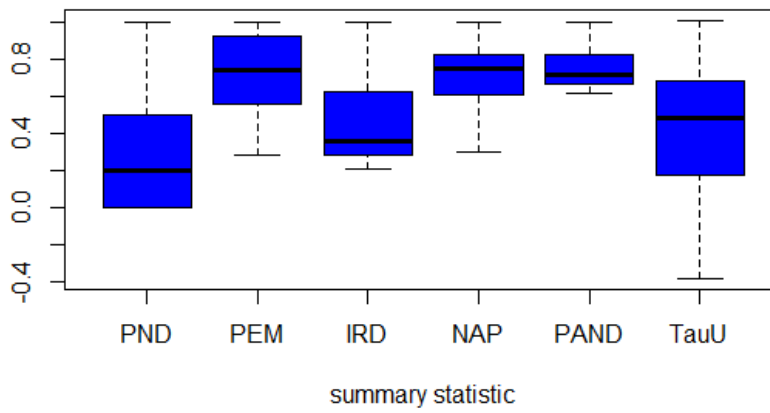

c.

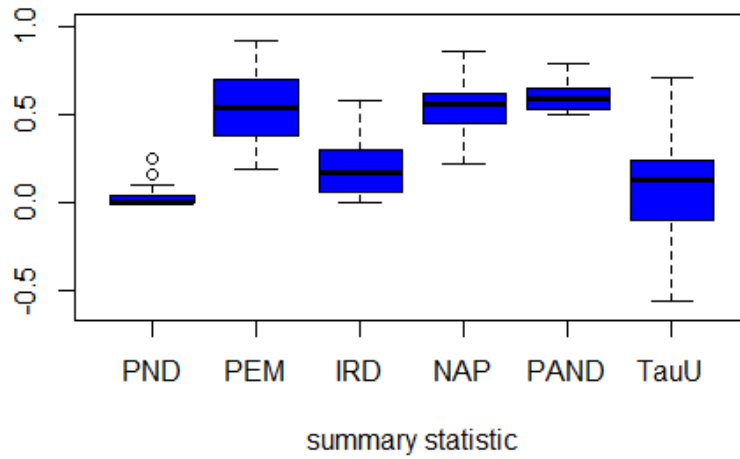

d.

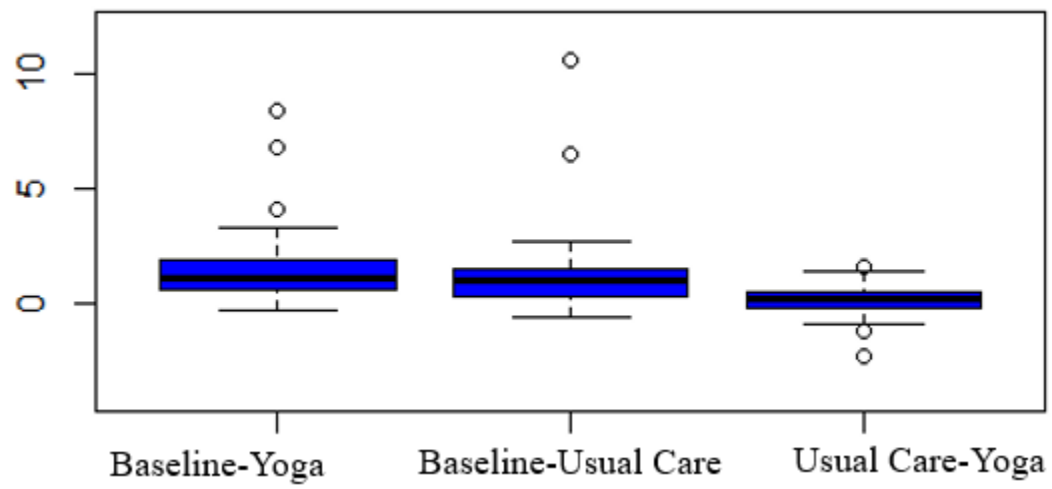

Supplement: Appendix B [file NIHMS1882450-supplement-Appendix_B.pdf]
