# Supplementary material for: Quantitative Synthesis of Personalized Trials Studies: Meta-Analysis of Aggregated Data Versus Individual Patient Data
Source: Harv Data Sci Rev. Author manuscript; Available in PMC 2023 Nov 24. (PMC10673630; doi:10.1162/99608f92.3574f1dc)
Supplement: Appendix A [file NIHMS1882450-supplement-Appendix_A.pdf]

Appendix A- Graphs

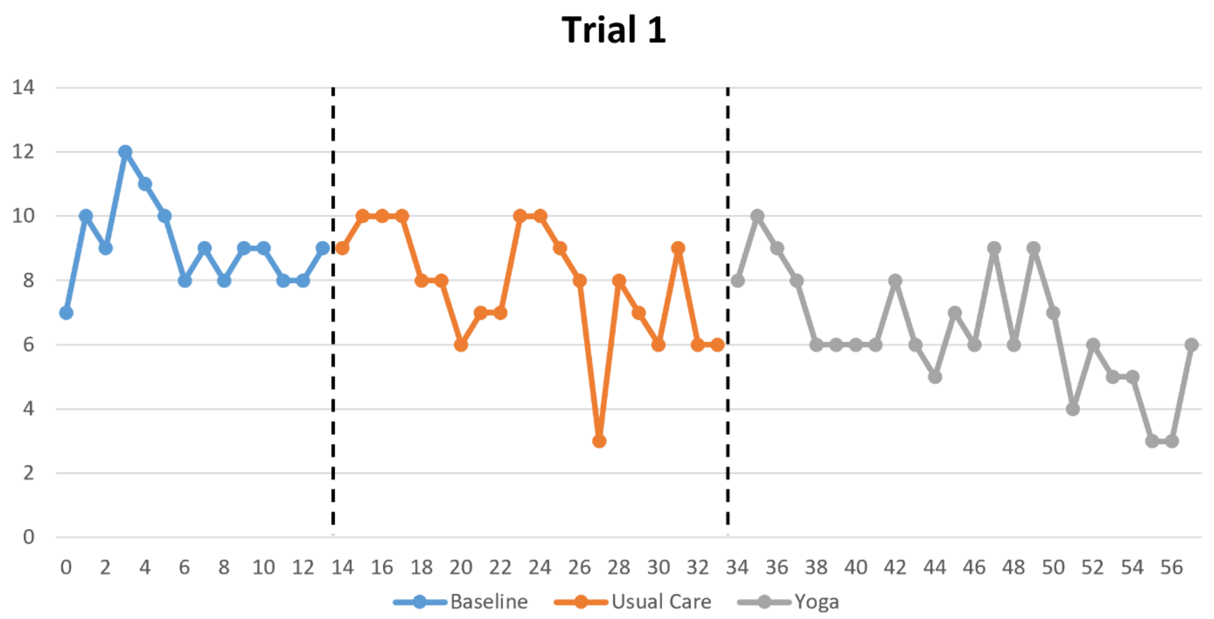

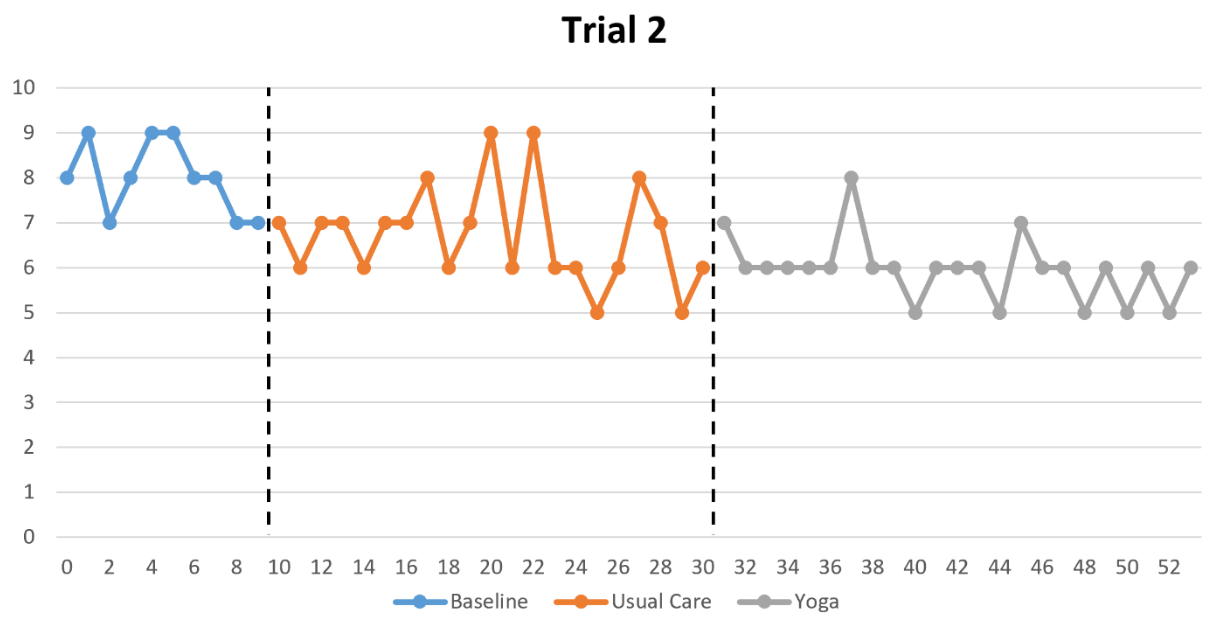

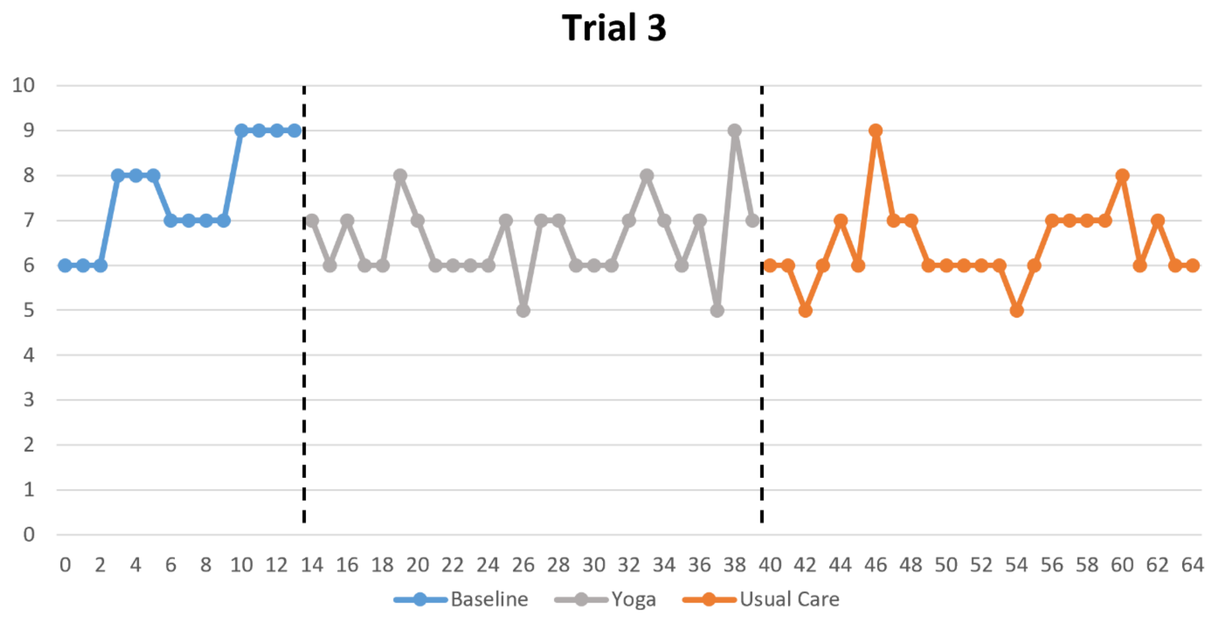
Trial 3
10
9
8
7
6
5
4
3
2
1
0
0 2 4 6 8 10 12 14 16 18 20 22 24 26 28 30 32 34 36 38 40 42 44 46 48 50 52 54 56 58 60 62 64
Baseline
Yoga
Usual Care

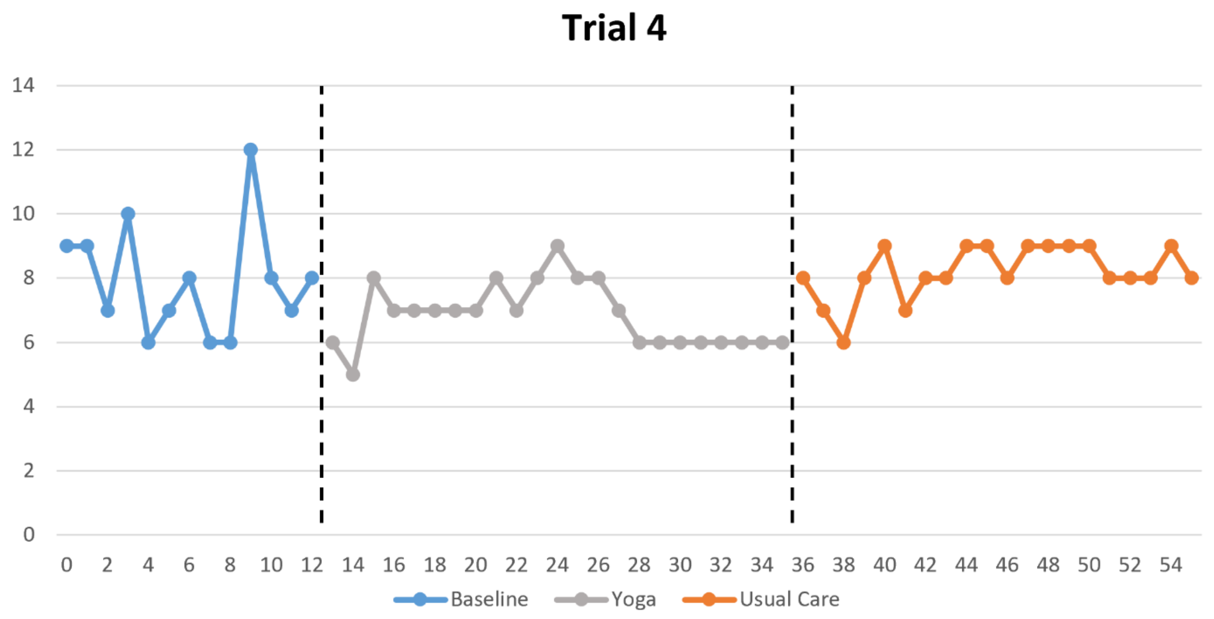
Trial 4
14
12
10
8
6
4
2
0
0 2 4 6 8 10 12 14 16 18 20 22 24 26 28 30 32 34 36 38 40 42 44 46 48 50 52 54
Baseline
Yoga
Usual Care

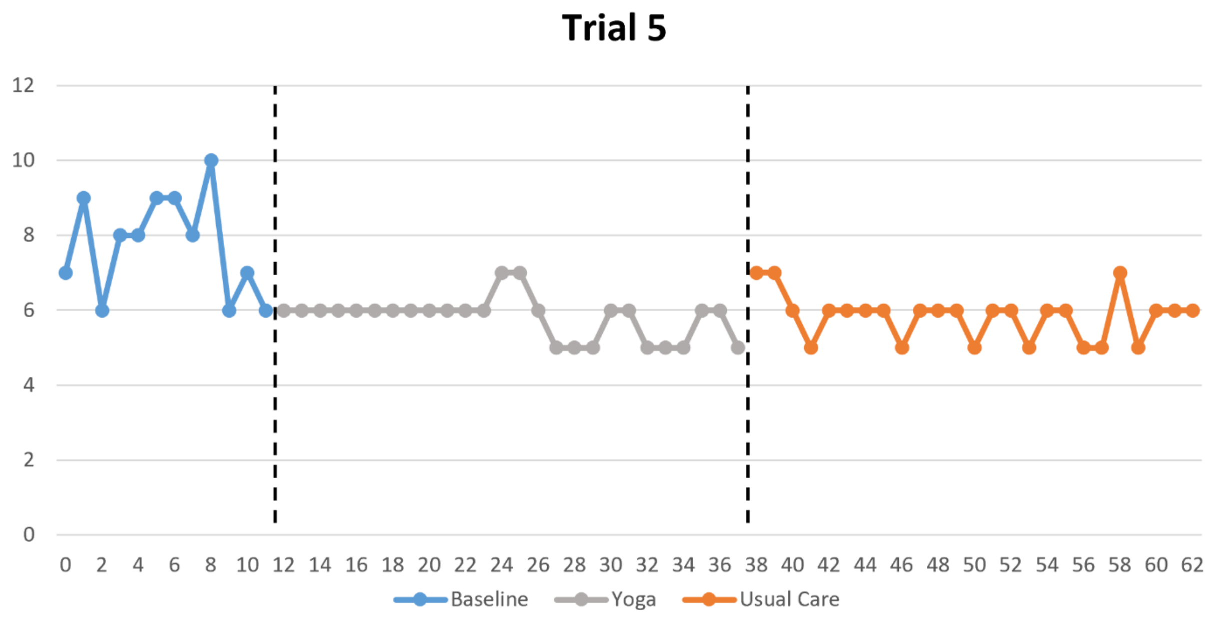
Trial 5
12
10
8
6
4
2
0
0 2 4 6 8 10 12 14 16 18 20 22 24 26 28 30 32 34 36 38 40 42 44 46 48 50 52 54 56 58 60 62
Baseline
Yoga
Usual Care

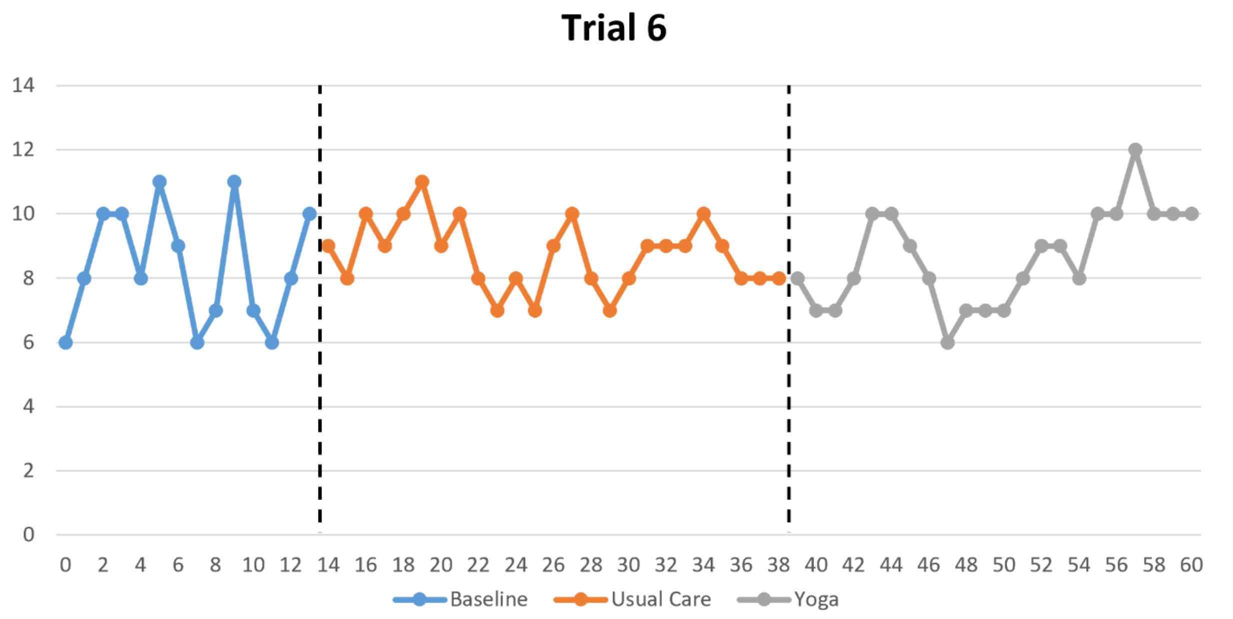
Trial 6
14
12
10
8
6
4
2
0
0 2 4 6 8 10 12 14 16 18 20 22 24 26 28 30 32 34 36 38 40 42 44 46 48 50 52 54 56 58 60
Baseline
Usual Care
Yoga

Trial 7

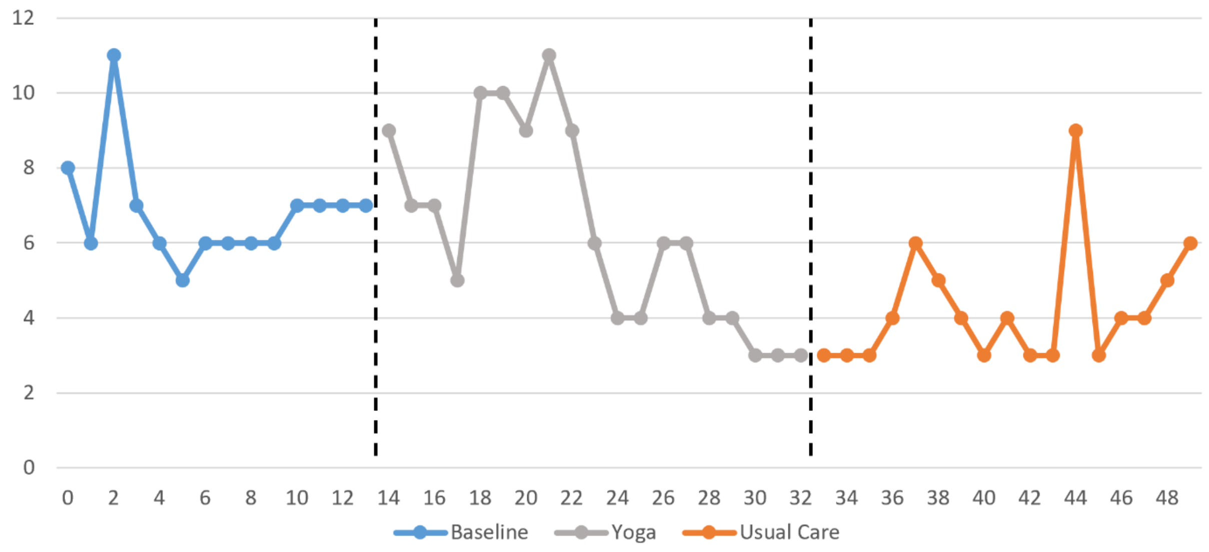

Trial 8

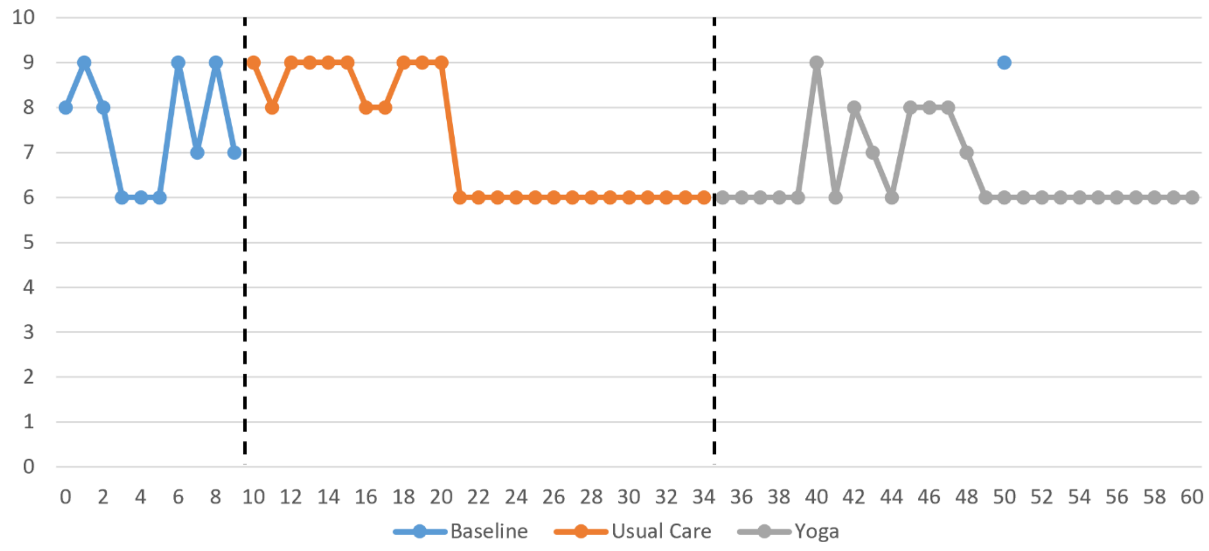

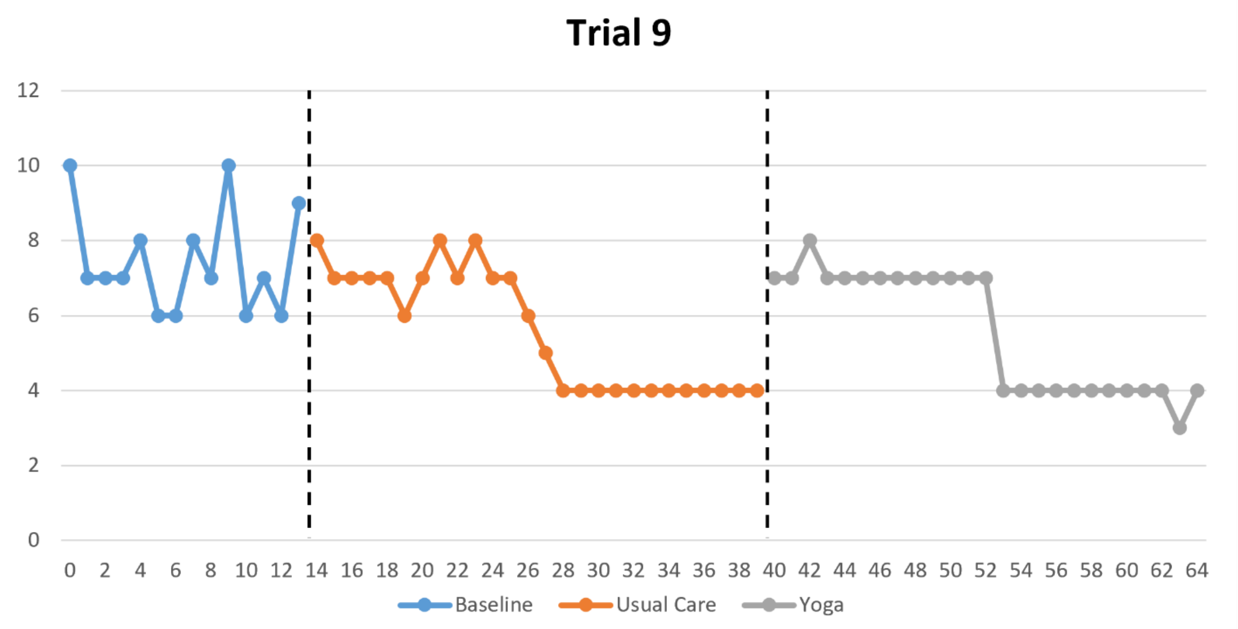
Trial 9
12
10
8
6
4
2
0
0 2 4 6 8 10 12 14 16 18 20 22 24 26 28 30 32 34 36 38 40 42 44 46 48 50 52 54 56 58 60 62 64
Baseline
Usual Care
Yoga

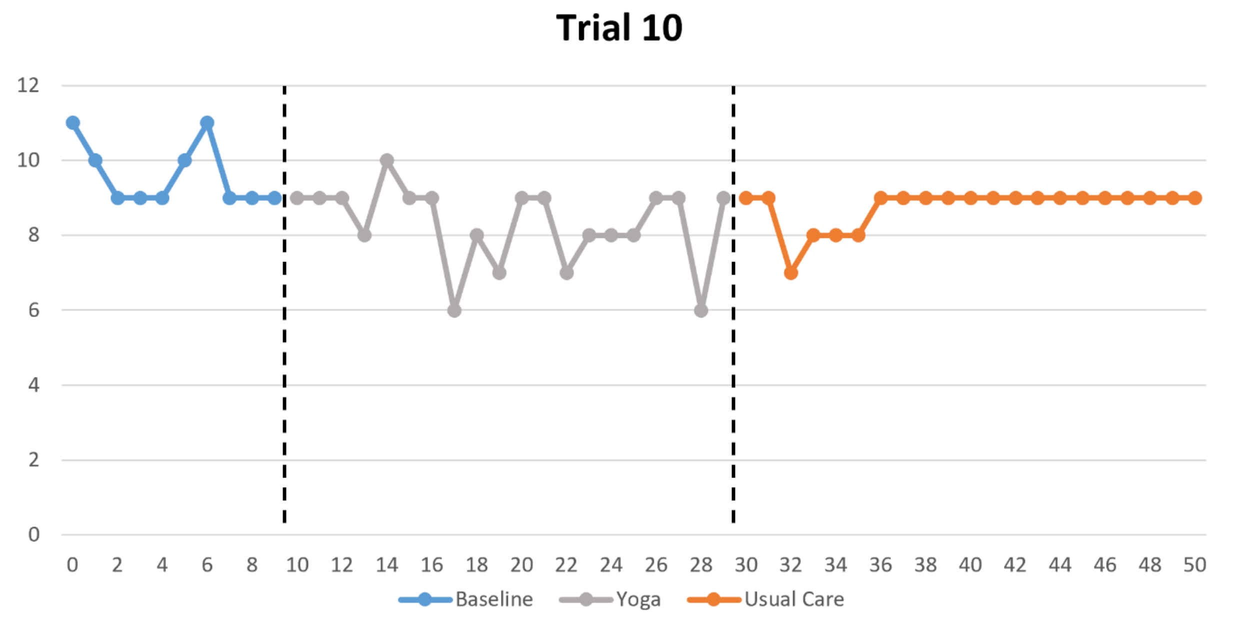
Trial 10
12
10
8
6
4
2
0
0 2 4 6 8 10 12 14 16 18 20 22 24 26 28 30 32 34 36 38 40 42 44 46 48 50
Baseline
Yoga
Usual Care

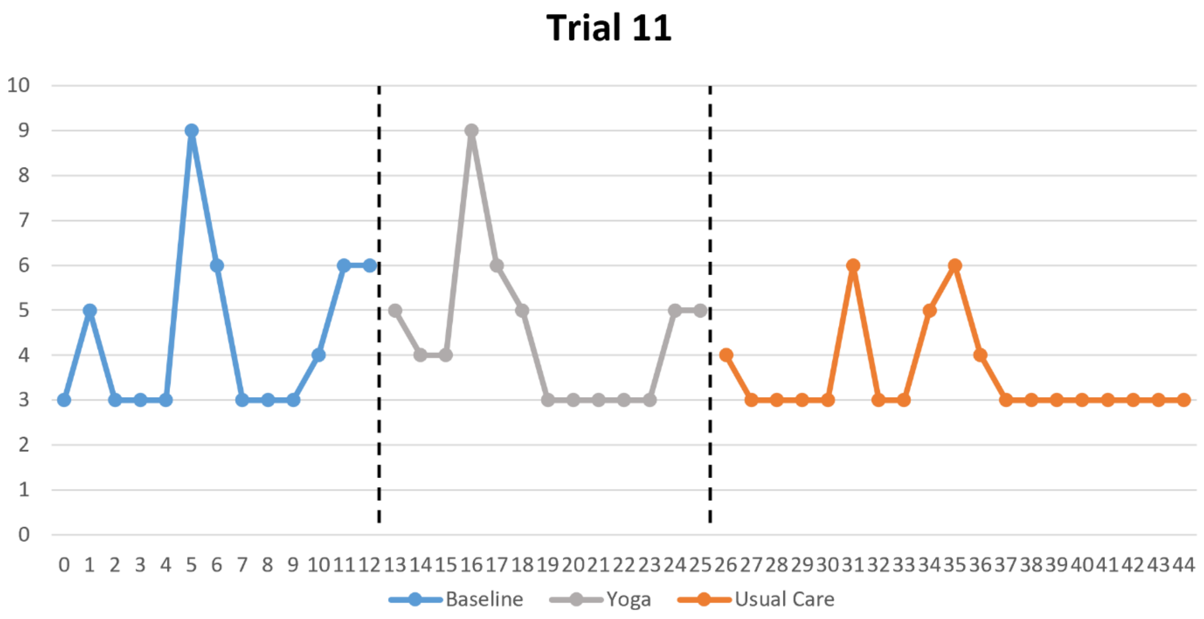
Trial 11
10
9
8
7
6
5
4
3
2
1
0
0 1 2 3 4 5 6 7 8 9 10 11 12 13 14 15 16 17 18 19 20 21 22 23 24 25 26 27 28 29 30 31 32 33 34 35 36 37 38 39 40 41 42 43 44
Baseline
Yoga
Usual Care

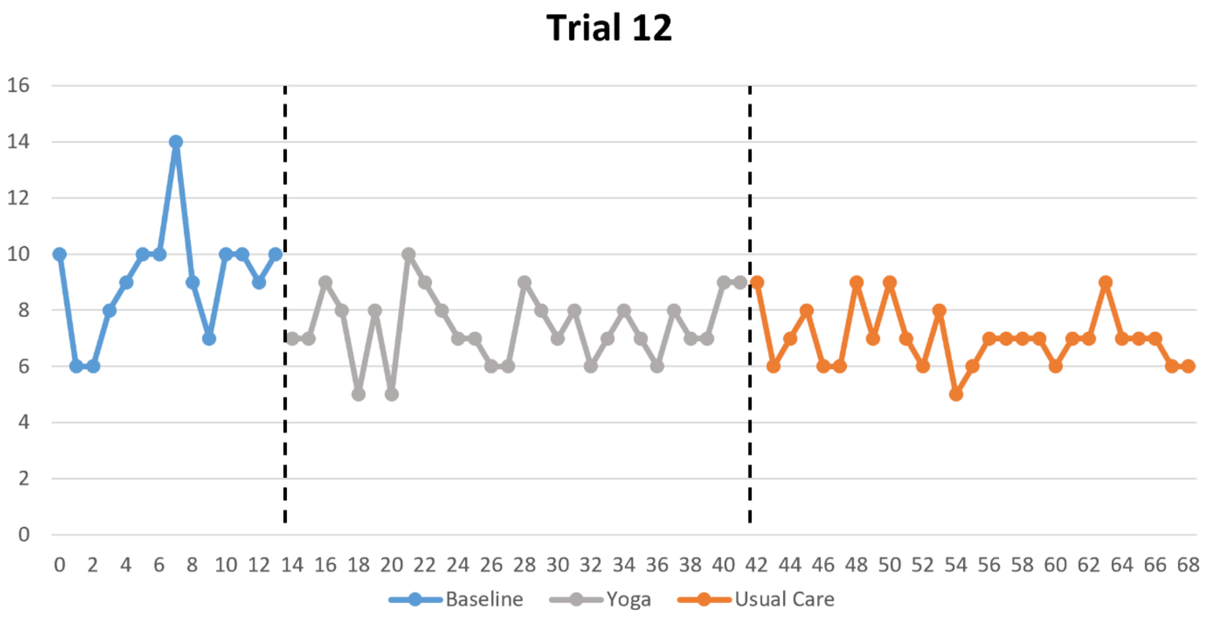
Trial 12
16
14
12
10
8
6
4
2
0
0 2 4 6 8 10 12 14 16 18 20 22 24 26 28 30 32 34 36 38 40 42 44 46 48 50 52 54 56 58 60 62 64 66 68
Baseline
Yoga
Usual Care

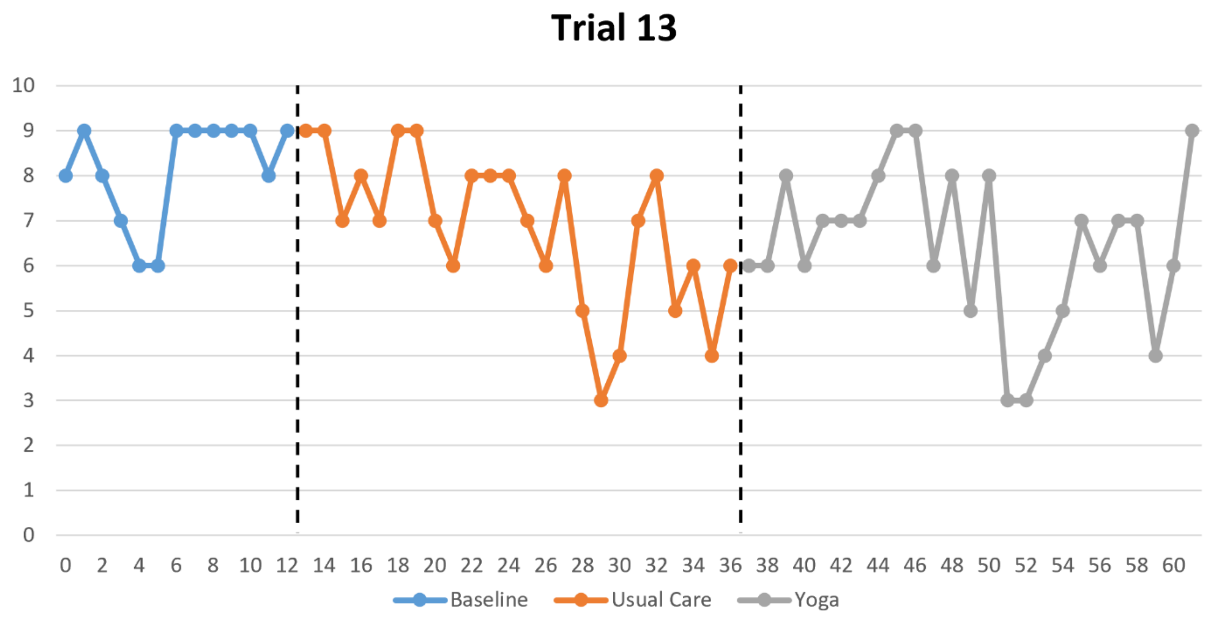
Trial 13
10
9
8
7
6
5
4
3
2
1
0
0 2 4 6 8 10 12 14 16 18 20 22 24 26 28 30 32 34 36 38 40 42 44 46 48 50 52 54 56 58 60
Baseline
Usual Care
Yoga

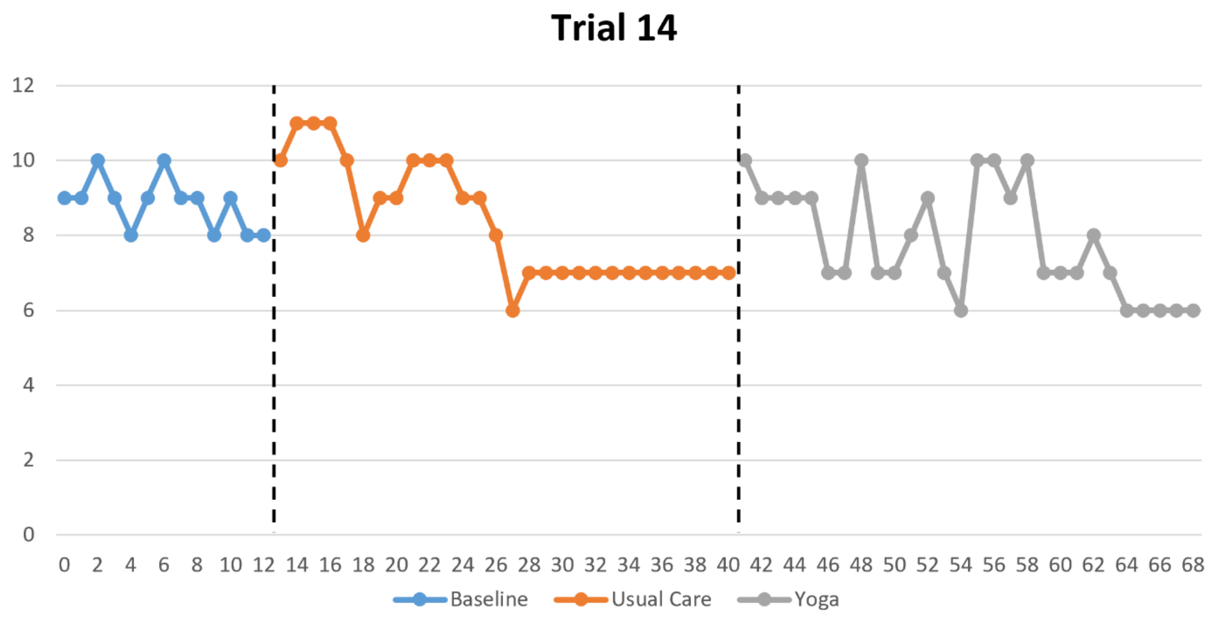
Trial 14
12
10
8
6
4
2
0
0 2 4 6 8 10 12 14 16 18 20 22 24 26 28 30 32 34 36 38 40 42 44 46 48 50 52 54 56 58 60 62 64 66 68
Baseline
Usual Care
Yoga

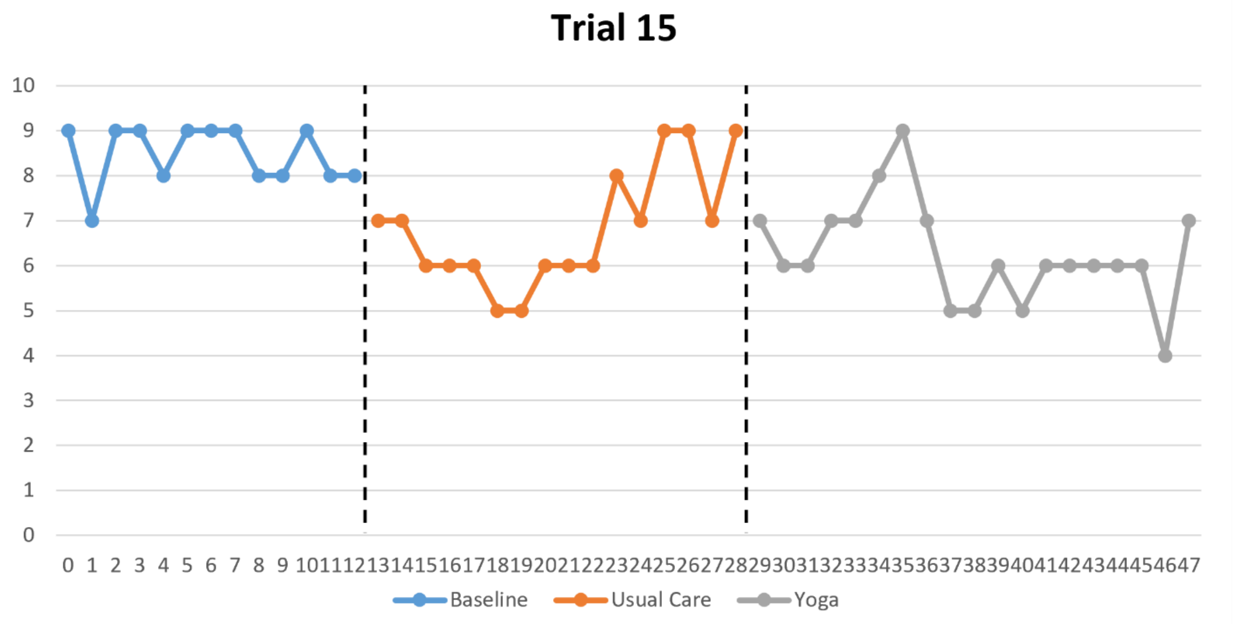
Trial 15
10
9
8
7
6
5
4
3
2
1
0
0 1 2 3 4 5 6 7 8 9 101112131415161718192021222324252627282930313233343536373839404142434445464 7
Baseline
Usual Care
Yoga

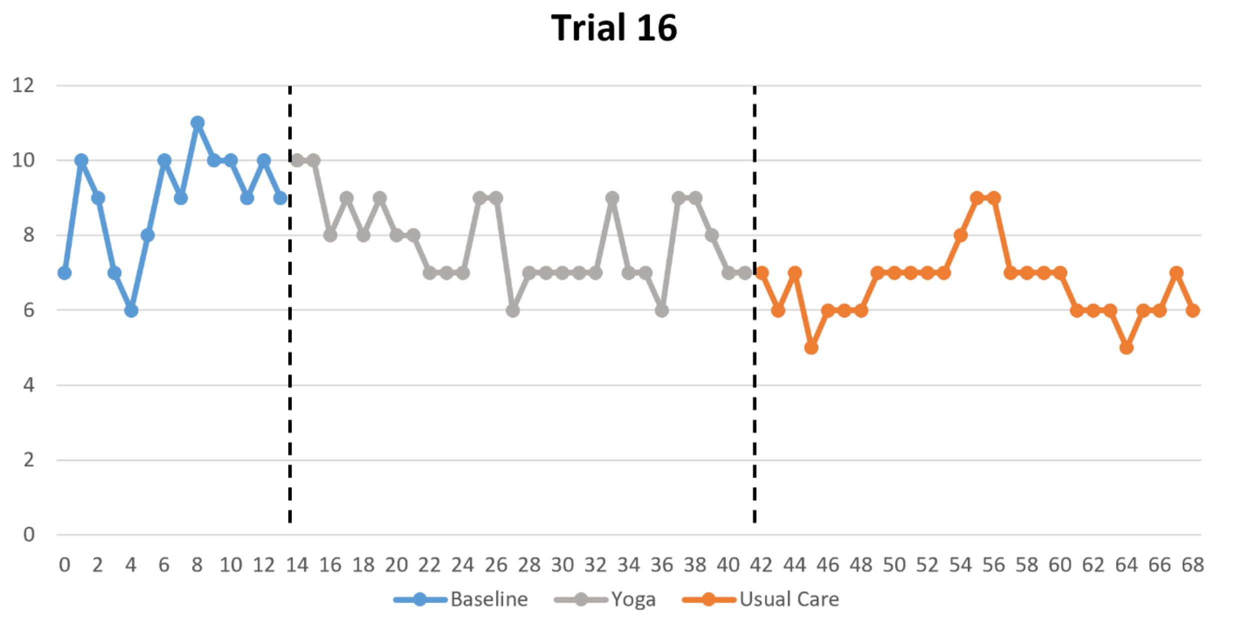
Trial 16
12
10
8
6
4
2
0
0 2 4 6 8 10 12 14 16 18 20 22 24 26 28 30 32 34 36 38 40 42 44 46 48 50 52 54 56 58 60 62 64 66 68
Baseline
Yoga
Usual Care

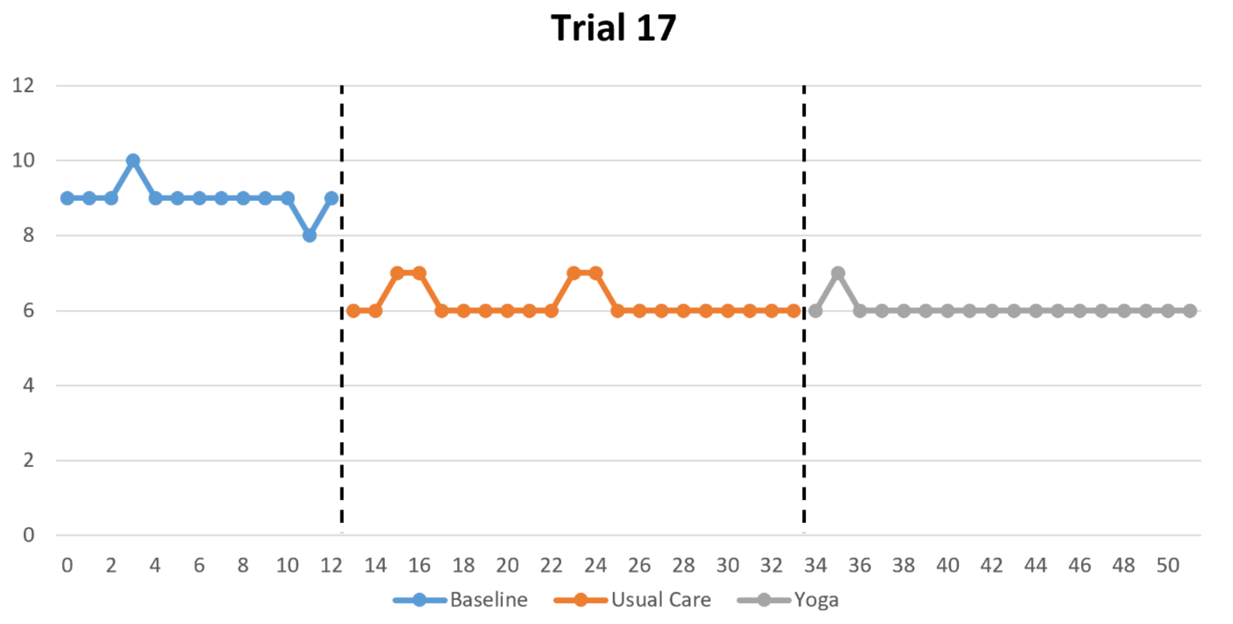

Trial 17
12
10
8
6
4
2
0
0 2 4 6 8 10 12 14 16 18 20 22 24 26 28 30 32 34 36 38 40 42 44 46 48 50
Baseline
Usual Care
Yoga

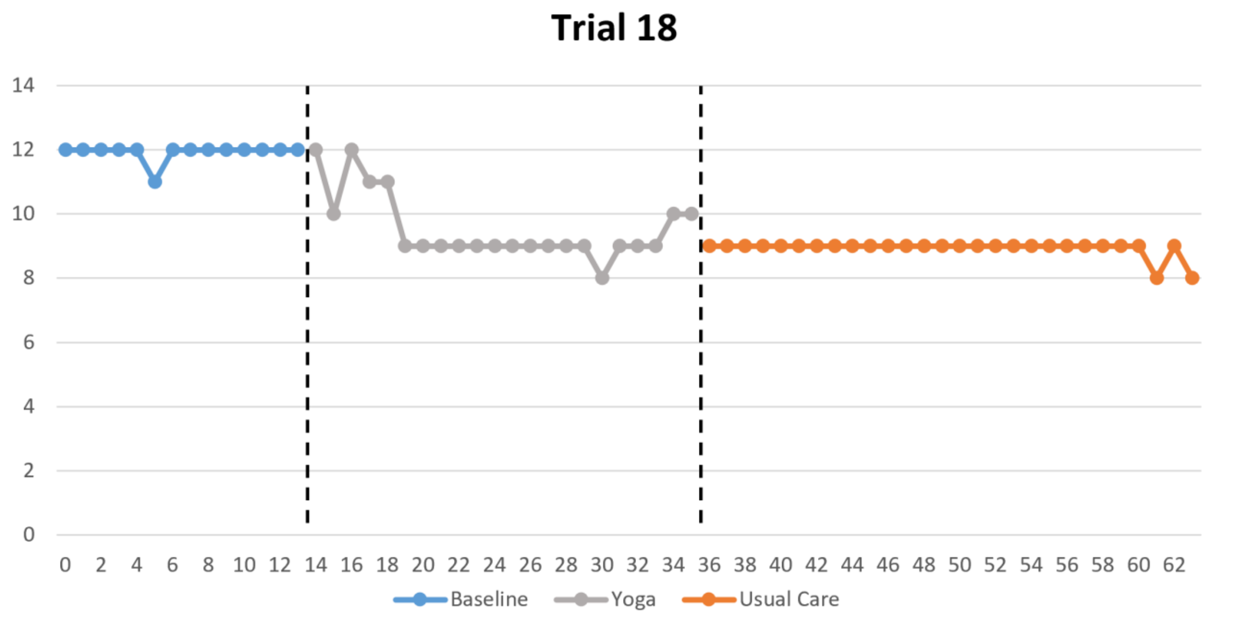

Trial 18
14
12
10
8
6
4
2
0
0 2 4 6 8 10 12 14 16 18 20 22 24 26 28 30 32 34 36 38 40 42 44 46 48 50 52 54 56 58 60 62
Baseline
Yoga
Usual Care

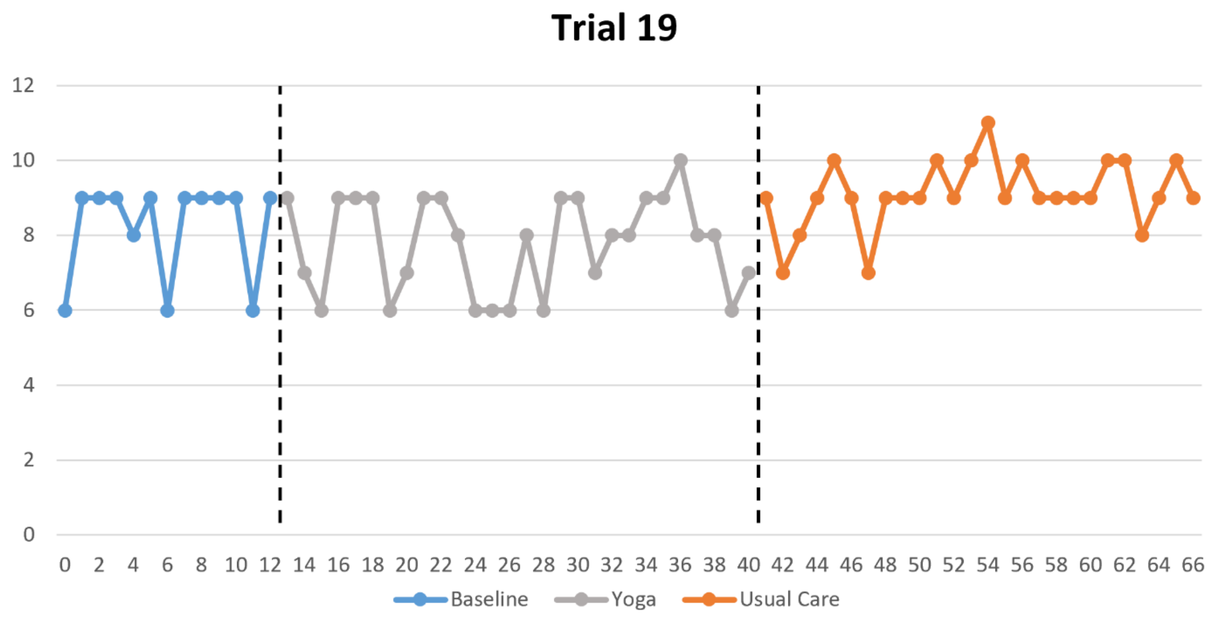
Trial 19
12
10
8
6
4
2
0
0 2 4 6 8 10 12 14 16 18 20 22 24 26 28 30 32 34 36 38 40 42 44 46 48 50 52 54 56 58 60 62 64 66
Baseline
Yoga
Usual Care

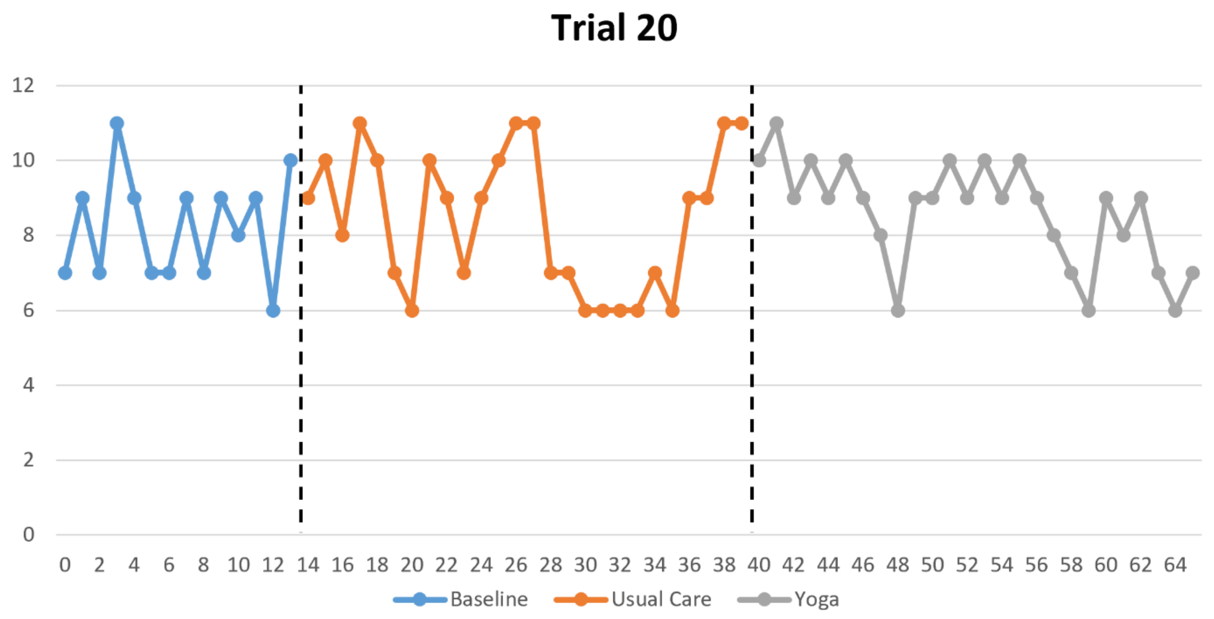
Trial 20
12
10
8
6
4
2
0
0 2 4 6 8 10 12 14 16 18 20 22 24 26 28 30 32 34 36 38 40 42 44 46 48 50 52 54 56 58 60 62 64
Baseline
Usual Care
Yoga

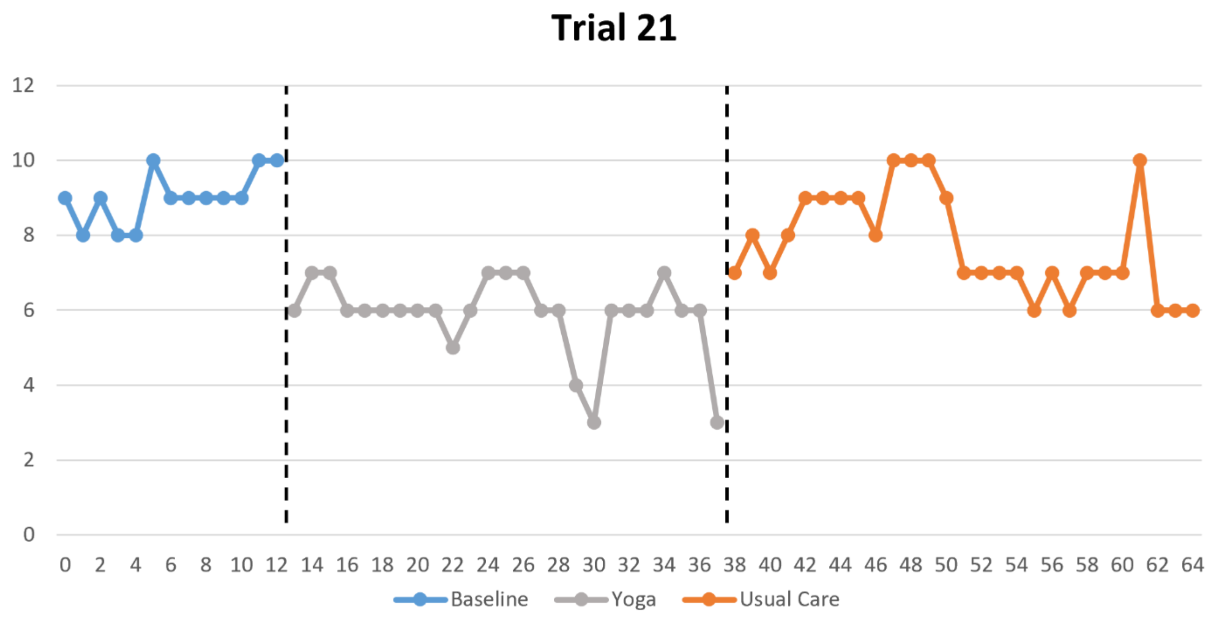
Trial 21
12
10
8
6
4
2
0
0 2 4 6 8 10 12 14 16 18 20 22 24 26 28 30 32 34 36 38 40 42 44 46 48 50 52 54 56 58 60 62 64
Baseline
Yoga
Usual Care

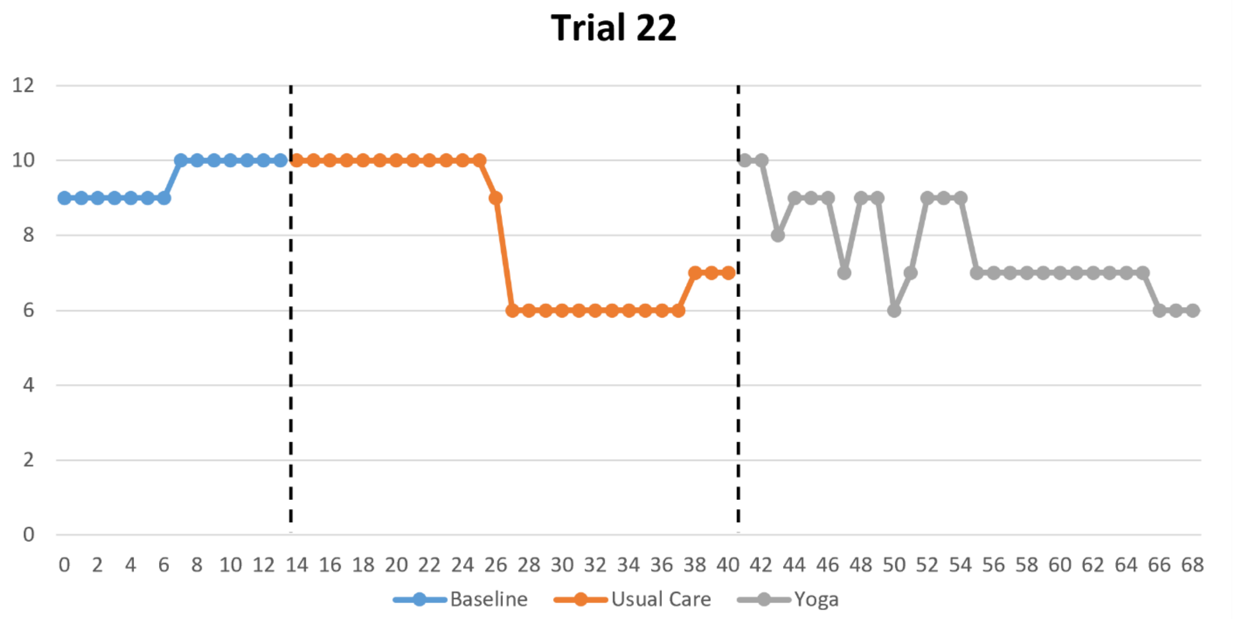
Trial 22
12
10
8
6
4
2
0
0 2 4 6 8 10 12 14 16 18 20 22 24 26 28 30 32 34 36 38 40 42 44 46 48 50 52 54 56 58 60 62 64 66 68
Baseline
Usual Care
Yoga

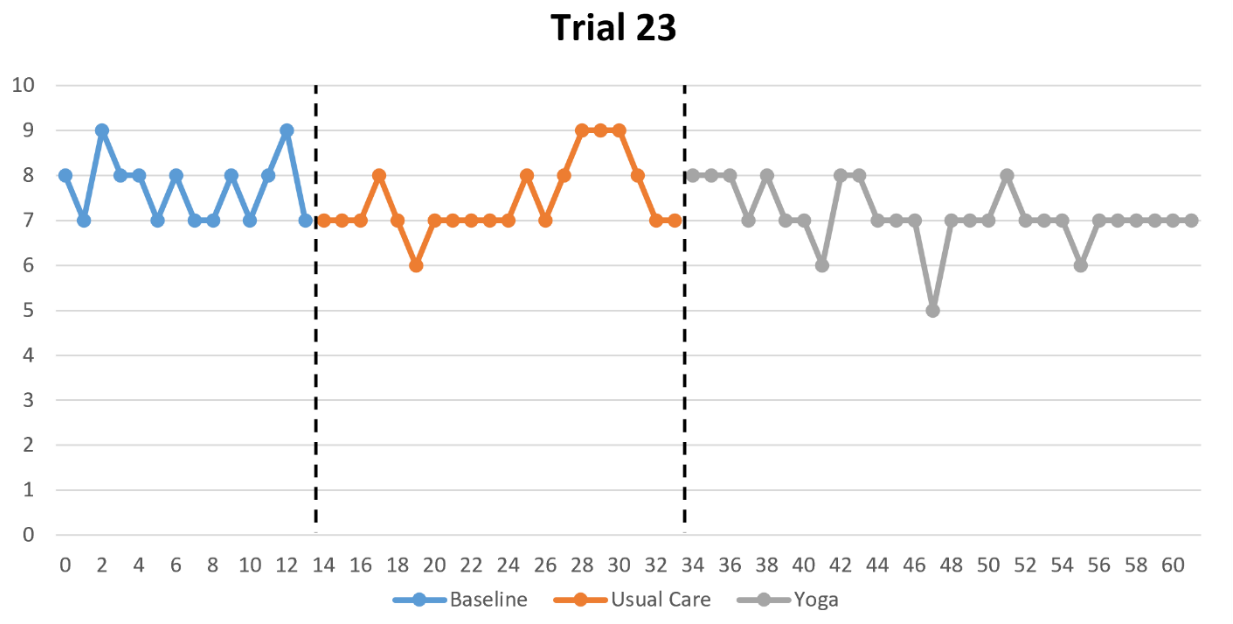
Trial 23
0 1 2 3 4 5 6 7 8 9 10
0 2 4 6 8 10 12 14 16 18 20 22 24 26 28 30 32 34 36 38 40 42 44 46 48 50 52 54 56 58 60
Baseline
Usual Care
Yoga

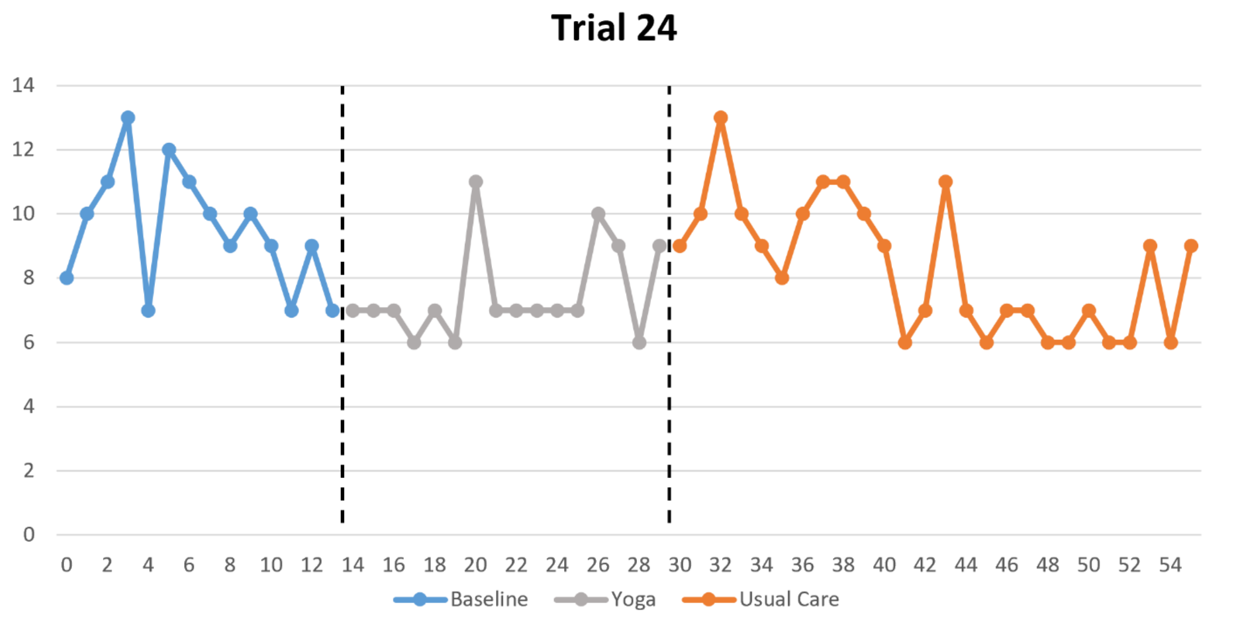
Trial 24
0 2 4 6 8 10 12 14
0 2 4 6 8 10 12 14 16 18 20 22 24 26 28 30 32 34 36 38 40 42 44 46 48 50 52 54
Baseline
Yoga
Usual Care

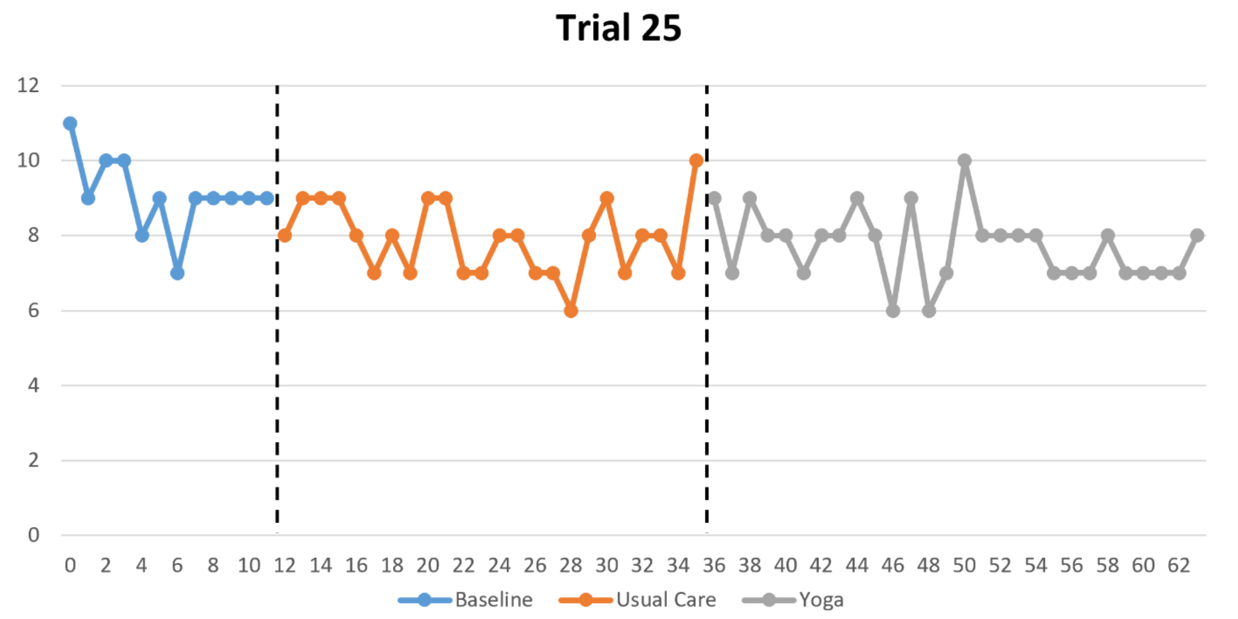
Trial 25
12
10
8
6
4
2
0
0 2 4 6 8 10 12 14 16 18 20 22 24 26 28 30 32 34 36 38 40 42 44 46 48 50 52 54 56 58 60 62
Baseline
Usual Care
Yoga

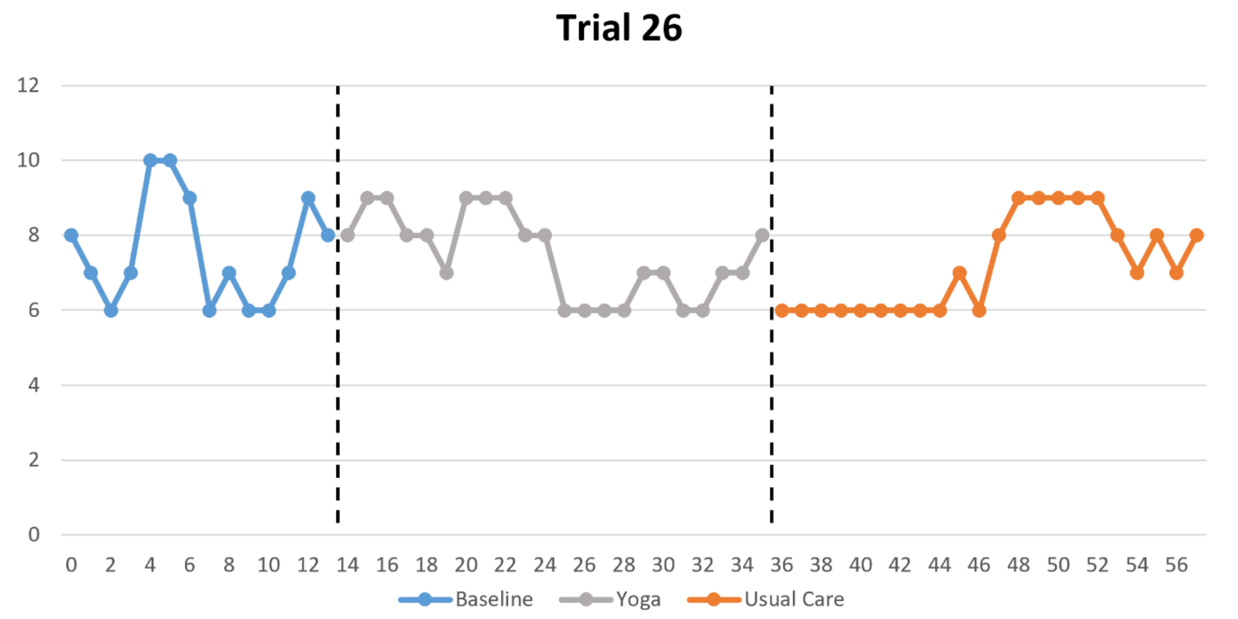
Trial 26
12
10
8
6
4
2
0
0 2 4 6 8 10 12 14 16 18 20 22 24 26 28 30 32 34 36 38 40 42 44 46 48 50 52 54 56
Baseline
Yoga
Usual Care
