# Supplementary material for: Quantitative Synthesis of Personalized Trials Studies: Meta-Analysis of Aggregated Data Versus Individual Patient Data
Source: Harv Data Sci Rev. Author manuscript; Available in PMC 2023 Nov 24. (PMC10673630; doi:10.1162/99608f92.3574f1dc)
Supplement: Appendix C [file NIHMS1882450-supplement-Appendix_C.docx]

**Appendix C- Models**

**Model 1**

$Y_{ij}=\beta_{0j}+\beta_{1j}Yoga {+ e}_{ij} \mathrm{with}e_{ij}\sim N\left( 0,\sigma_{e}^{2} \right)$ (1)

In Equation 1, $Y_{ij}$ refers to the outcome score (pain level) at measurement occasion *i*, for subject *j*. $\beta_{0j}$ indicates the average pain level during the baseline phase. $\beta_{1j}$ indicates the average change between the baseline phase and the Yoga intervention. $e_{ij}$ indicates the normally disturbed within-participant residual. The within-participant residuals, $e_{ij}$’s, are homogeneous, independent and normally distributed.$Yoga$ is a dummy variable, equaling 0 during baseline and 1 during the intervention.

$\left\{ \begin{aligned} \beta_{0j}=\theta_{0}+u_{0j} \\ \beta_{1j}=\theta_{1}+u_{1j} \end{aligned} \right.$ with $\left[ \begin{aligned} \begin{aligned} u_{0j} \\ u_{1j} \end{aligned} \end{aligned} \right]\sim MVN\left( 0, \zeta\right)$ , and $e_{ij}\sim N(0,\sigma_{e}^{2}$) (2)

In Equation 2, $\theta_{0}$ indicates the mean baseline level across trials. $\theta_{1}$indicates the mean intervention level across trials. Referring to the same equation, $u_{0j}$ and $u_{1j}$indicate the deviations from the across trials parameters. The deviations are assumed to be multivariate normally distributed.

**Model 2**

$Y_{ij}=\beta_{0j}+\beta_{1j}{Time}_{ij} {+\beta_{2j}{Yoga}_{ij}+\beta_{3j}{{Time'}_{ij}\times Yoga}_{ij}+ e}_{ij} \mathrm{with}e_{ij}\sim N\left( 0,\sigma_{e}^{2} \right)$ (3)

In Equation 3, $Y_{ij}$ refers to the outcome score (pain level) at measurement occasion *i*, for subject *j*. $\beta_{0j}$ indicates the average pain level during the baseline phase. $\beta_{1j}$ indicates the time trend during the baseline phase. $\beta_{2j}$ indicates the average change between the baseline phase and the Yoga intervention. $\beta_{3j}$ indicates the average change in trend between the baseline phase and the Yoga intervention phase. $e_{ij}$ indicates the normally disturbed within-participant residual. The within-participant residuals, $e_{ij}$’s, are homogeneous, independent and normally distributed. ${Time'}_{ij}$ indicates the time of the interaction term, centered around the first session of the intervention phase. $Yoga$ is a dummy variable, equaling 0 during baseline and 1 during the intervention.

$\left\{ \begin{aligned} \beta_{0j}=\theta_{0}+u_{0j} \\ \beta_{1j}=\theta_{1}+u_{1j} \\ \beta_{2j}=\theta_{2}+u_{2j} \\ \beta_{3j}=\theta_{3}+u_{3j} \end{aligned} \right.$ with $\left[ \begin{aligned} \begin{aligned} u_{0j} \\ u_{1j} \end{aligned} \\ u_{2j} \\ u_{3j} \end{aligned} \right]\sim MVN\left( 0, \zeta\right)$ , and $e_{ij}\sim N(0,\sigma_{e}^{2}$) (4)

In Equation 4, $\theta_{0}$ indicates the mean baseline level across trials. $\theta_{1}$indicates the mean baseline trend across trials. $\theta_{2}$ indicates the mean Yoga intervention level across trials. $\theta_{3}$ indicates the mean change in trend between the baseline and Yoga intervention phase across trials. Referring to the same equation, $u_{0j}$, $u_{1j}$, $u_{2j}$, and $u_{3j}$indicate the deviations from the across trials parameters. The deviations are assumed to be multivariate normally distributed.

**Model 3a**

$Y_{ij}=\beta_{0j}{+\beta_{1j}{Yoga}_{ij}+ \beta_{2j}{Usual Care}_{ij}+e}_{ij} \mathrm{with}e_{ij}\sim N\left( 0,\sigma_{e}^{2} \right)$ (5)

In Equation 5,$Yoga$ and $Usual Care$ are dummy variables, equaling 0 during baseline or 1 during the intervention. $Y_{ij}$ refers to the outcome score (pain level) at measurement occasion *i*, for trial *j*. $\beta_{0j}$ indicates the average pain level during the baseline phase. $\beta_{1j}$ indicates the average change between the baseline phase and the Yoga intervention. $\beta_{2j}$ indicates the average change between the baseline phase and the Usual Care intervention. $e_{ij}$ indicates the normally disturbed within-participant residual. The within-participant residuals, $e_{ij}$’s, are homogeneous, independent and normally distributed

$\left\{ \begin{aligned} \beta_{0j}=\theta_{0}+u_{0j} \\ \beta_{1j}=\theta_{1}+u_{1j} \\ \beta_{2j}=\theta_{2}+u_{2j} \end{aligned} \right.$ with $\left[ \begin{aligned} \begin{aligned} u_{0j} \\ u_{1j} \end{aligned} \\ u_{2j} \end{aligned} \right]\sim MVN\left( 0, \zeta\right)$ , and $e_{ij}\sim N(0,\sigma_{e}^{2}$) (6)

In Equation 6, $\theta_{0}$ indicates the mean baseline level across trials. $\theta_{1}$ indicates the mean Yoga intervention level across trials. $\theta_{2}$ indicates the mean Usual Care intervention level across trials. Referring to the same equation, $u_{0j}$, $u_{1j}$, and $u_{2j}$,indicate the deviations from the across trials parameters. The deviations are assumed to be multivariate normally distributed.

**Model 3b**

$Y_{ij}=\beta_{0j}{+\beta_{1j}{Intervention}_{ij}+ \beta_{2j}{\Delta Intervention}_{ij}+e}_{ij} \mathrm{with}e_{ij}\sim N\left( 0,\sigma_{e}^{2} \right)$ (7)

In Equation 7,$Intervention$ and $\Delta Intervention$ are dummy variables. $Intervention$ equals 0 during baseline and 1 during the intervention phases. $\Delta Intervention$ equals 0 during the baseline and the Usual Care phase, and 1 during the Yoga intervention phase. $Y_{ij}$ refers to the outcome score (pain level) at measurement occasion *i*, for trial *j*. $\beta_{0j}$ indicates the average pain level during the baseline phase. $\beta_{1j}$ indicates the average change between the baseline phase and the Usual Care phase. $\beta_{2j}$ indicates the average change between the Usual Care phase and the Yoga phase. $e_{ij}$ indicates the normally disturbed within-participant residual. The within-participant residuals, $e_{ij}$’s, are homogeneous, independent and normally distributed

$\left\{ \begin{aligned} \beta_{0j}=\theta_{0}+u_{0j} \\ \beta_{1j}=\theta_{1}+u_{1j} \\ \beta_{2j}=\theta_{2}+u_{2j} \end{aligned} \right.$ with $\left[ \begin{aligned} \begin{aligned} u_{0j} \\ u_{1j} \end{aligned} \\ u_{2j} \end{aligned} \right]\sim MVN\left( 0, \zeta\right)$ , and $e_{ij}\sim N(0,\sigma_{e}^{2}$) (8)

In Equation 8, $\theta_{0}$ indicates the mean baseline level across trials. $\theta_{1}$ indicates the overall average change between baseline and Usual Care. $\theta_{2}$ indicates the mean difference between Usual Care and Yoga. Referring to the same equation, $u_{0j}$, $u_{1j}$, and $u_{2j}$,indicate the deviations from the across trials parameters. The deviations are assumed to be multivariate normally distributed.
